# The oral nucleoside analogue inhibitor VV251 effectively inhibits coinfection by respiratory syncytial virus and influenza A virus

**DOI:** 10.1128/jvi.00006-26

**Published:** 2026-04-21

**Authors:** Ruxue Zhang, Xiaoqin Jian, Yumin Zhang, Yuan Sun, Mengwei Xu, Junyuan Cao, Guanghui Tian, Li Xiang, Jiangshan Shen, Gengfu Xiao, Tianwen Hu, Leike Zhang

**Affiliations:** 1 State Key Laboratory of Virology, Wuhan Institute of Virology, Chinese Academy of Sciences, Center for Biosafety Mega-Science, Wuhan, Hubei, China; 2 University of Chinese Academy of Sciences 74519, Beijing, China; 3 Hubei Jiangxia Laboratory, Wuhan, Hubei, China; 4 Vigonvita Shanghai Co., Ltd., Shanghai, China; 5 State Key Laboratory of Drug Research, Shanghai Institute of Materia Medica, Chinese Academy of Science, Shanghai, China; University of North Carolina at Chapel Hill, Chapel Hill, North Carolina, USA

**Keywords:** VV251, coinfection treatment, influenza A virus, respiratory syncytial virus

## Abstract

Respiratory syncytial virus (RSV) and influenza virus are the principal contributors to respiratory tract infections worldwide. Clinical treatment of RSV or influenza A virus (IAV) is limited and may be postponed because of misdiagnosis or coinfection with multiple pathogens. Here, we described VV251, the prodrug of 4′-fluorouridine, a novel oral nucleoside analog that exhibits potent inhibitory activity against RSV laboratory strains and clinical isolates, as well as IAV, *in vitro*. The EC_50_ values of VV251 against these viruses ranged from nanomolar to low micromolar. Moreover, VV251 significantly reduced the viral titer of RSV A2 or IAV PR8 in a BALB/c infection model. Importantly, we further established a simultaneous RSV/IAV coinfection model to investigate the antiviral activity of VV251. Oral administration of VV251 once daily significantly inhibited viral replication and decreased the mortality of RSV/IAV-coinfected mice. Our findings establish VV251 as a broad-spectrum drug candidate for the treatment of RSV, IAV, and RSV/IAV coinfection.

**IMPORTANCE:**

Respiratory syncytial virus (RSV) and influenza virus are the two predominant causative agents of acute respiratory tract infections, leading to a substantial number of hospitalizations and thousands of deaths annually. The cost of treating disease caused by infection with RSV and influenza viruses is a huge financial burden on the world. We have identified a nucleotide analog with favorable pharmacokinetic properties and antiviral activity against RSV and influenza A virus (IAV) during mono- or coinfection in a mouse model. This compound has the potential to be used for the treatment of RSV, IAV, and RAV/IAV coinfection.

## INTRODUCTION

Respiratory syncytial virus (RSV) and influenza virus are the two predominant causative agents of acute respiratory tract infections, leading to a substantial number of hospitalizations and thousands of deaths annually (1–3). RSV, which belongs to the *Pneumoviridae* family, is a contagious human pathogen that causes severe respiratory tract infections globally, particularly in infants, the elderly, and immunocompromised populations (4, 5). To date, ribavirin is the only nonmonoclonal drug approved by the Food and Drug Administration (FDA) for RSV treatment, but its clinical application is limited because of its limited efficacy and teratogenic attributes (6). Influenza virus, which belongs to the *Orthomyxoviridae* family, infects all age groups and is estimated to contribute to approximately 290,000–650,000 annual respiratory deaths globally (7–9). Currently, six antivirals against influenza virus are FDA approved, with Japan and China additionally authorizing favipiravir (T-705) for clinical use (10–14). However, viral resistance has continually emerged to some of these drugs, highlighting an urgent need for novel agents with alternative mechanisms of action. RSV and influenza virus induce overlapping clinical symptoms, presenting challenges and consuming substantial time for medical professionals in diagnosis and treatment decision-making.

Clinically, coinfections with multiple pathogens are prevalent, constituting 10%–30% of respiratory viral infections and thereby complicating the diagnostic and treatment process (15–17). Coinfection with RSV and influenza virus has been shown to exacerbate disease severity (18, 19). Moreover, Haney et al. confirmed that coinfection with RSV and influenza A virus (IAV) generates infectious hybrid virus particles, which can utilize the RSV fusion glycoprotein to evade anti-IAV neutralizing antibodies and infect cells lacking IAV receptors, thereby altering viral antigenicity and enhancing viral transmission among cells (20). Previous studies have focused predominantly on the interactions between viruses and the influence of coinfection on disease severity, but relatively few studies have evaluated the antiviral activity of broad-spectrum compounds in viral coinfection models. Hence, the development of safe and efficacious therapeutic strategies for RSV and influenza virus infections, particularly within the context of viral coinfections, remains a significant unmet medical need.

Nucleoside analogs play crucial roles in the field of clinical antivirals. They are the preferred agents for treating viral diseases such as viral hepatitis (21). The exploration of broad-spectrum nucleoside antivirals for the treatment of various RNA viruses has increased, revealing a promising avenue. 4′-Fluorouridine (4′-FlU), a novel broad-spectrum nucleoside analog targeting the viral RdRp, has significant antiviral activity against multiple viruses, including RSV, SARS-CoV-2, and influenza virus (22, 23). Mechanistic studies revealed that its antiviral effect is mainly mediated by chain termination, as shown through primer extension assays (22, 23). However, 4′-FlU has poor chemical stability under water and phosphate buffer, which may significantly limit its application (24, 25).

By evaluating the stability and antiviral activity of multiple 4′-FlU prodrugs, we identified VV251, a prodrug with three acetyl groups on the ribose moiety and a nicotinyloxymethyl group linked to imine nitrogen on the base moiety, which exhibits enhanced chemical stability. In this study, preliminary *in vitro* studies indicated that VV251 exhibits potent inhibitory efficacy against both RSV and IAV, with EC_50_ values ranging from nanomolar to low micromolar. *In vivo* models further confirmed the ability of the compound to significantly reduce viral titers in RSV and IAV mono- and simultaneous coinfection models. These findings not only establish VV251 as a candidate for the treatment of respiratory viral infections but also suggest its potential in confronting the increasingly severe challenge of viral coinfections.

## RESULTS

### Assessment of compound stability and pharmacokinetic characteristics

**Fig 1:**
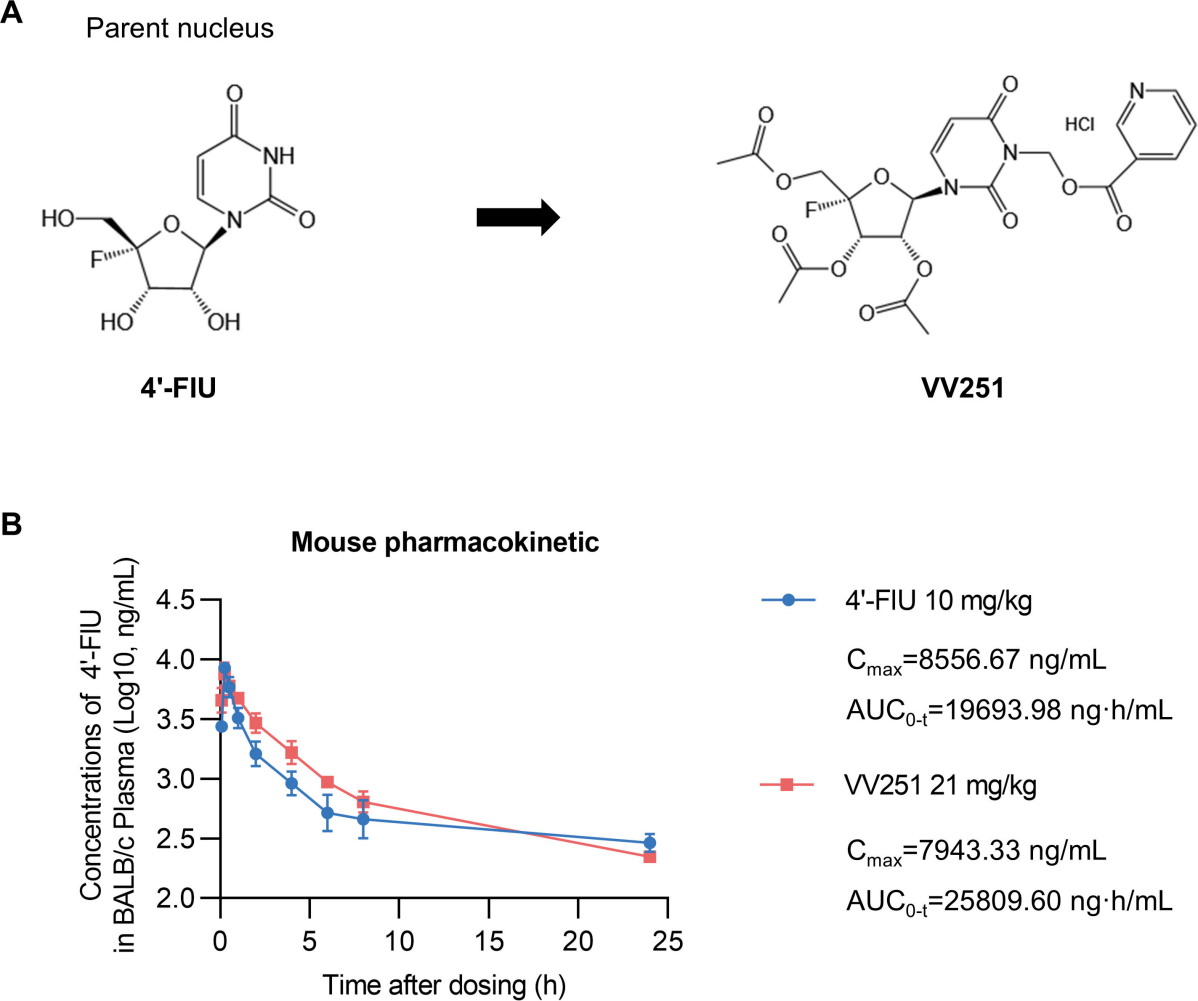
Pharmacokinetic properties of VV251 in mice. (**A**) Chemical structures of 4′-FlU and VV251. (**B**) The pharmacokinetic properties of orally administered equimolar doses of 4′-FlU and its prodrug VV251 were systematically characterized in the plasma of BALB/c mice. The data are shown as the means, with error bars indicating the SEM (*n* = 3 mice per group).

Previous studies have indicated that 4′-FlU is a broad-spectrum candidate for clinical trials (22, 23). However, the chemical stability of 4′-FlU is poor, as determined through changes in its content via high performance liquid chromatography (HPLC) in pH 2.0 and 4.0 buffer at 30°C after 7 days, which may significantly limit its application (24). Therefore, by employing the prodrug strategy, we identified the compound VV251, a 4′-FlU prodrug with three acetyl groups on the ribose moiety and a nicotinyloxymethyl group linked to imine nitrogen on the base moiety (Fig. 1A). To directly compare the stability of VV251 and 4′-FlU, we conducted stability assays under acidic (pH 1.5, simulating gastric fluid) and neutral (pH 6.8, simulating intestinal fluid) conditions. The results showed that after 24 h, 4′-FlU was almost completely degraded under acidic conditions and lost approximately 80% of its purity under neutral conditions (Table S1). In contrast, VV251 showed significantly higher stability, with purity reductions of only about 30% and 20% in acidic and neutral solutions, respectively (Table S1).

A head-to-head pharmacokinetic comparison of 4′-FlU and its prodrug VV251 was conducted in BALB/c mice following oral administration of equimolar doses. The results demonstrated that VV251 administration led to rapid appearance of 4′-FlU in plasma, achieving peak concentration (*C*_max_) approximately 0.25 h after dosing, which was consistent with that observed following direct oral administration of 4′-FlU (Fig. 1B). Although the half-life (*T*_1/2_) of VV251 was approximately half that of 4′-FlU, it still exhibited a favorable pharmacokinetic profile with a *T*_1/2_ of 9.20 h (Table S3). Moreover, treatment with VV251 resulted in higher plasma concentrations of the active metabolite 4′-FlU compared to direct administration of 4′-FlU. The area under the curve (AUC_0*–t*_) for VV251 was 25,809.60 ng·h/mL, compared to 19,693.98 ng·h/mL for 4′-FlU (Fig. 1B), indicating enhanced systemic exposure. Previous studies have disclosed elevated 4′-FlU concentrations in the lung tissue of both mice and guinea pigs, potentially indicating associations with diseases related to the respiratory tract (23, 26). These results suggest that VV251 may have antiviral potential and warrant further study.

### VV251 displays broad-spectrum antiviral activity against RSV *in vitro*

**Fig 2:**
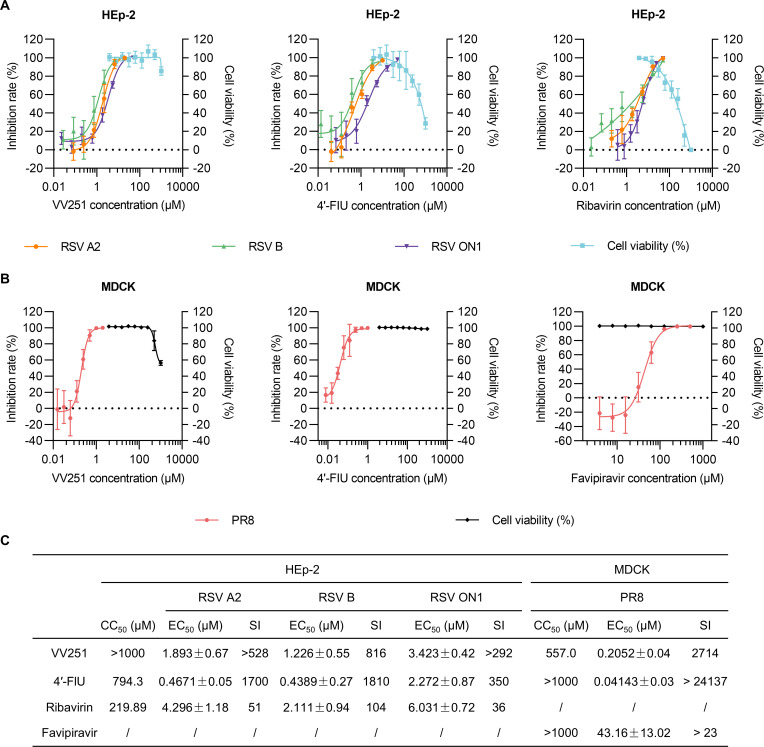
The broad-spectrum antiviral activity of VV251 *in vitro*. (**A**) Dose‒response assay of VV251, 4′-FlU, and ribavirin against RSV (RSV A2, RSV B, and RSV ON1 strains) and cytotoxicity profiles of VV251, 4′-FlU, and ribavirin in HEp-2 cells. (**B**) Activity of VV251, 4′-FlU, and favipiravir in inhibiting influenza A virus (PR8) and cytotoxicity profiles of VV251, 4′-FlU, and favipiravir in MDCK cells. (**C**) The half-maximum effective concentration (EC_50_) and half-maximal cytotoxic concentration (CC_50_) of the compounds (VV251, 4′-FlU, ribavirin, and favipiravir) in HEp-2 cells or MDCK cells. EC_50_ and CC_50_ values based on four-parameter variable slope regression modeling are provided. This analysis allows the calculation of the selectivity index (SI), which is defined as the ratio of the CC_50_ to the EC_50_. The data are presented as the mean values of independent experiments (*n* = 3) ± standard deviation (SD).

To evaluate the antiviral activity of VV251, we initially evaluated the inhibitory effects of VV251 and 4′-FlU on the RSV A2 strain in HEp-2 cells. Both VV251 and 4′-FlU exerted potent inhibitory effects on RSV A2 replication, with half-maximal effective concentration (EC_50_) of 1.893 μM and 0.4671 μM, respectively (Fig. 2A). Moreover, the cytotoxicity of VV251 was negligible in HEp-2 cells, with a half-maximal cytotoxic concentration (CC_50_) exceeding 1,000 μM. In contrast, the CC_50_ value of 4′-FlU was determined to be 794.3 μM. Consequently, when calculated as the ratio of CC_50_ to EC_50_, the selectivity indices (SI) of VV251 and 4′-FlU were 528 and 1,700, respectively (Fig. 2A and C). In addition, the antiviral activities of VV251 and 4′-FlU were further evaluated in other RSV-permissive cell lines, namely A549 cells and 16HBE cells. Specifically, in A549 cells, the EC_50_ values were determined to be 1.784 μM for VV251 and 0.1333 μM for 4′-FlU, while in 16HBE cells, the corresponding EC_50_ values were 0.3580 μM and 0.04939 μM, respectively (Fig. S1). Subsequently, we evaluated the antiviral activity of VV251 and 4′-FlU against RSV B. In HEp-2 cells, both compounds exhibited potent activity against RSV B, with EC_50_ values of 1.226 μM and 0.4389 μM, respectively (Fig. 2A and C). Recently, a new RSV ON1 genotype, containing a repeat of 72 nucleotides in the c-terminal region of the attached (G) glycoprotein, was first identified in Canada in 2010 (27). This genotype has rapidly replaced the NA1 genotype and has become the dominant genotype prevalent in many countries around the world (28–30). A clinical strain of RSV with the ON1 genotype was isolated from nasopharyngeal swabs and purified for experimental investigation. As shown in Fig. 2A, VV251 was active against RSV ON1, a low-passage RSV A clinical isolate, with an average EC_50_ value of 3.423 μM. The EC_50_ value of 4′-FlU was 2.272 μM. Since the highest concentration of 4′-FlU metabolized from 10 mg/kg oral VV251 in mice is higher than the cellular EC_50_ concentration (antiviral activity of VV251 against RSV A2 strain), further exploration of the antiviral activity of VV251 *in vivo* is feasible.

### VV251 exhibits antiviral activity against influenza A virus *in vitro*

To assess the antiviral activity of VV251 against influenza virus, we measured the antiviral activity of VV251 and 4′-FlU in MDCK cells in parallel. Dose–response experiments revealed that VV251 effectively suppressed the influenza A virus strain A/Puerto Rico/8/34 (PR8), achieving an EC_50_ of 0.2052 μM (Fig. 2B and C). Notably, this compound showed relatively low cytotoxicity in MDCK cells, with a CC_50_ value of 557.0 μM (Fig. 2B and C), resulting in a high selectivity index of 2714. In contrast, 4′-FlU displayed more pronounced antiviral activity, with an EC_50_ of 0.04143 μM, a CC_50_ exceeding 1,000 μM, and a calculated SI of 24,137. Guided by the pharmacokinetics of VV251 and tissue distribution data of 4′-FlU, as well as the results of a dose‒response study against IAV, the appropriate dose was chosen as the optimal dosage for further exploration of the efficacy of VV251 against IAV *in vivo*.

### VV251 reduces viral replication in RSV A2-infected BALB/c mice

**Fig 3:**
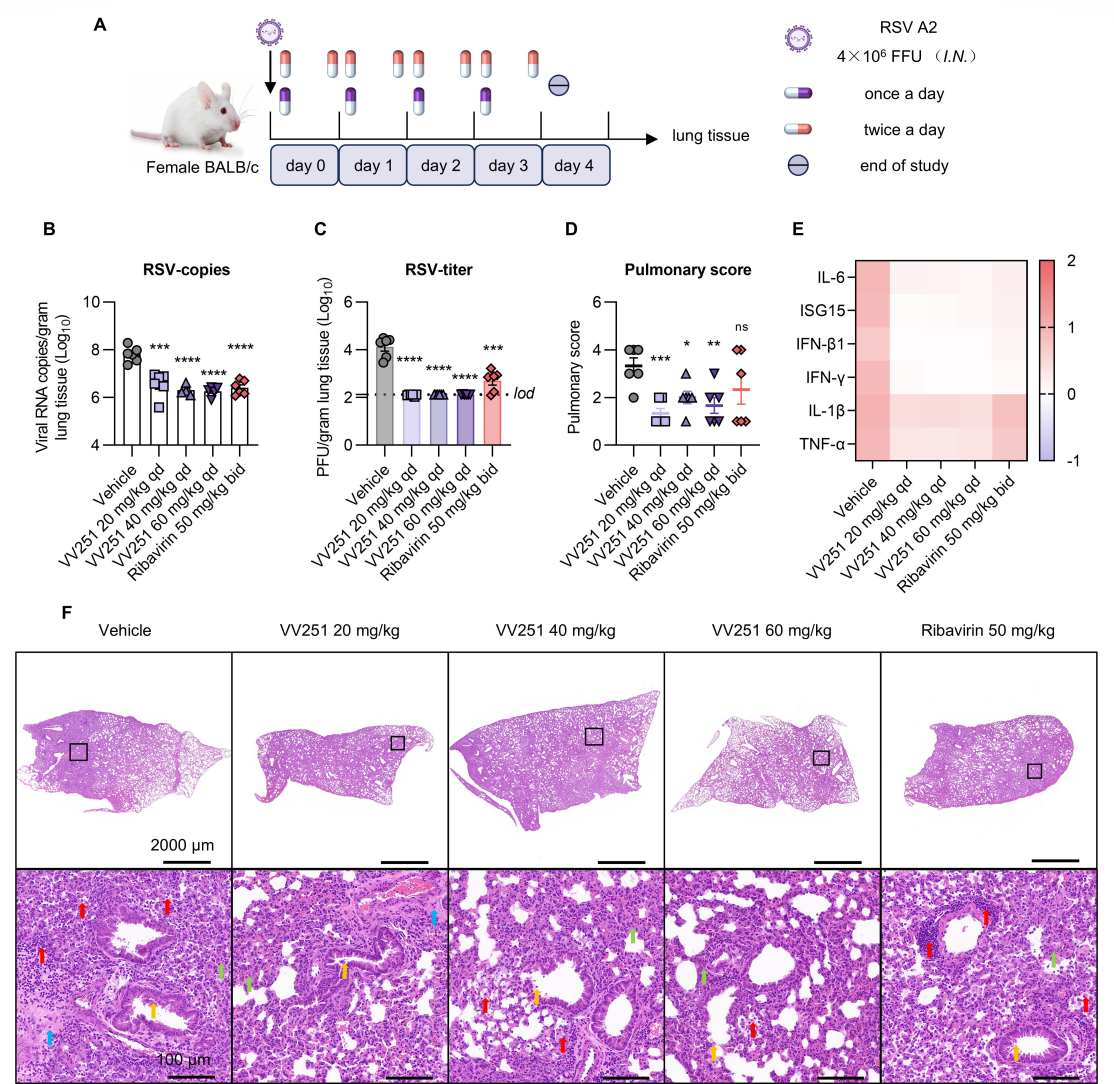
Therapeutic efficacy of VV251 against RSV A2 in a BALB/c mouse model. (**A**) Schematic of the efficacy study in BALB/c mice. (**B**) Determination of viral RNA copies targeting F genes in the lungs collected on day 4 by real-time fluorescence quantitative PCR (*n* = 6). (**C**) Infections RSV A2 titers in the lungs of the mice were measured at 4 dpi by immuno-plaque assays (*n* = 6). The line represents the limit of detection (*lod*). (**D**) The lung tissue pathological score was evaluated at 4 dpi in therapeutically treated mice compared with that in vehicle-treated mice (*n* = 6). (**E**) Cytokine gene expression was measured in the lungs on day 4 by a relative quantitative method (*n* = 6). GAPDH was used as the internal reference gene. (**F**) Representative images of histopathological changes in the lungs of RSV A2-infected BALB/c mice at 4 dpi. The whole-lung tissue scan images and magnified views of the boxed regions for each image are shown. Red arrows indicate inflammatory cell infiltration, orange arrows indicate epithelial cell shedding, green arrows indicate thickening of the alveolar wall, and blue arrows indicate thickening of perivascular connective tissue. Scale bars indicate 2,000 μm and 100 μm. The symbols represent individual values, and the error bars indicate the SEM (*n* = 6). Statistical significance compared to the vehicle group was analysed by an unpaired Student’s *t* test. *P < 0.05; **P < 0.01; ***P < 0.001; ****P < 0.0001; and ns, not significant.

To assess the antiviral activity of VV251 *in vivo*, we initially evaluated the therapeutic efficacy of VV251 against RSV A2 in BALB/c mice, as shown in Fig. 3A. The mice were intranasally challenged with RSV A2 and subsequently orally administered VV251 or ribavirin at 1‒2 h post-infection (hpi). Ribavirin was administered at a dose of 50 mg/kg as a positive control. The ribavirin group was administered ribavirin twice daily (bid, 12 h interval), whereas all the other groups were administered the treatment once a day (qd). At 4 days post-infection (dpi), which corresponds to the peak of viral titer, the lung tissue of the mice was harvested for further analysis. Compared with the vehicle control, VV251 significantly inhibited RSV A2 replication, as measured by the viral copy number and viral titer (Fig. 3B and C). Even the lowest dose (20 mg/kg) of VV251 was able to reduce the viral copy load by approximately 1.25log_10_ and decrease the viral titer below the detection limit (Fig. 3B and C). Gene expression analysis of cytokines (including IL-6, ISG15, IFN-β1, IFN-γ, IL-1β, and TNF-α) revealed that all treatment groups exhibited varying degrees of suppression in mRNA levels compared to the vehicle group (Fig. 3E; Fig. S4). Notably, the three dosage groups of VV251 demonstrated statistically significant inhibition across multiple inflammatory markers. Collectively, these results imply that VV251 has significant potential as an efficacious antiviral agent against RSV A2 *in vivo*.

**Fig 4:**
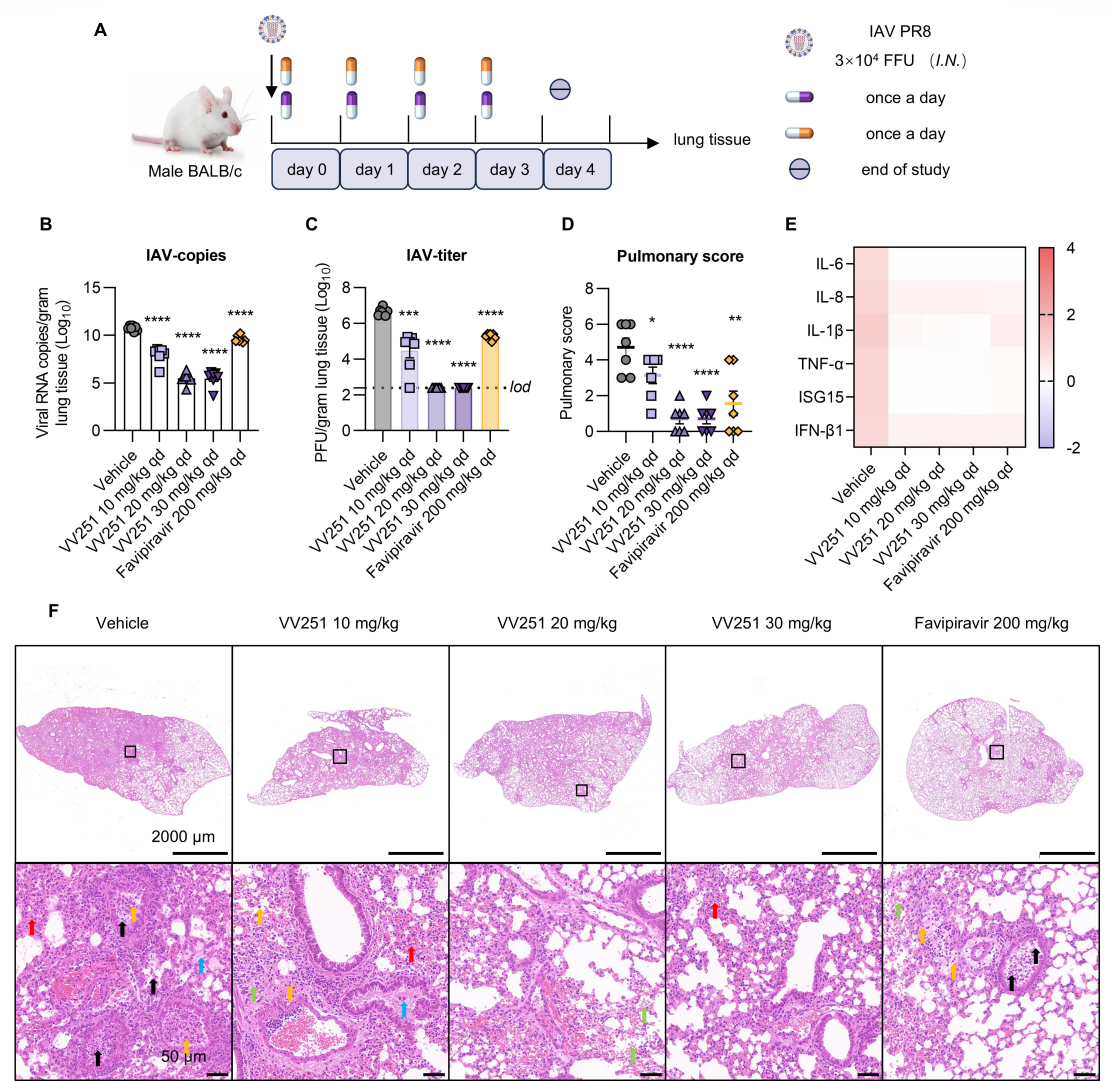
*In vivo* efficacy of VV251 in BALB/c mice infected with the IAV PR8 strain. (**A**) Schematic of the experimental design for the treatment of BALB/c mice. (**B**) Determination of the number of viral RNA copies targeting the HA gene in the lungs collected on day 4 by real-time fluorescence quantitative PCR (*n* = 7). (**C**) Determination of viral titers in the lungs harvested on day 4 by immuno-plaque assays (*n* = 7). The line represents the limit of detection (*lod*). (**D**) Lung tissue pathological scores at 4 dpi are shown for vehicle-, VV251-, and favipiravir-treated mice (*n* = 7). (**E**) Cytokine gene expression was measured in the lungs on day 4 (*n* = 7). GAPDH was used as the internal reference gene. (**F**) Histopathological analysis of the lung tissues from the vehicle, VV251 (10 mg/kg, 20 mg/kg, or 30 mg/kg), and favipiravir 200 mg/kg groups. Red arrows indicate inflammatory cell infiltration, orange arrows indicate epithelial cell shedding, green arrows indicate thickening of the alveolar wall, blue arrows indicate thickening of perivascular connective tissue, and black arrows indicate cell necrosis, nuclear fragmentation, and condensation. Scale bars indicate 2,000 μm and 50 μm. For panels B to D, each symbol represents data for one mouse, and the line is the mean. The data for viral copy numbers, viral titers, and lung tissue pathological scores compared to the vehicle group were statistically analysed with Student’s *t* test. *P < 0.05; **P < 0.01; ***P < 0.001; ****P < 0.0001; and ns, not significant.

### *In vivo* efficacy of VV251 against IAV PR8 in the BALB/c model

In addition, we evaluated the anti-PR8 efficacy of orally administered VV251 (10 mg/kg, 20 mg/kg, or 30 mg/kg, qd) in BALB/c mice, as shown in Fig. 4A. Favipiravir was administered at a dose of 200 mg/kg once a day as the positive control, as it has been previously reported to reduce the viral load of influenza virus in mice (31, 32). The mice in the vehicle group experienced body weight loss exceeding 20% at 4 dpi, whereas those in the VV251 group exhibited only 3.3% body weight loss until 4 dpi (Fig. S5). The lowest dose of VV251 (10 mg/kg) significantly reduced the number of viral copies and viral titers in the lungs by approximately 2.8log_10_ and 2.2log_10_, respectively (Fig. 4B and C). When the mice were treated with VV251 at the middle and highest doses, a reduction of more than 5.2log_10_ in the viral RNA copy number was observed, and the viral titers were below the detection limit at 4 dpi (Fig. 4B and C). A histopathological analysis of the lungs of the mice was performed at 4 dpi. Compared with that in the vehicle group, lung damage in all the mice treated with VV251 and favipiravir was significantly alleviated (Fig. 4D and F). PR8 infection triggered host inflammatory responses in mice; however, treatment with VV251 decreased the expression of inflammatory cytokines (Fig. 4E; Fig. S6). Taken together, our findings show that oral treatment with VV251 significantly reduces viral loads and ameliorates lung pathology in PR8-infected BALB/c mice.

### VV251 protects mice from lethal RSV A2 and IAV PR8 coinfection

**Fig 5:**
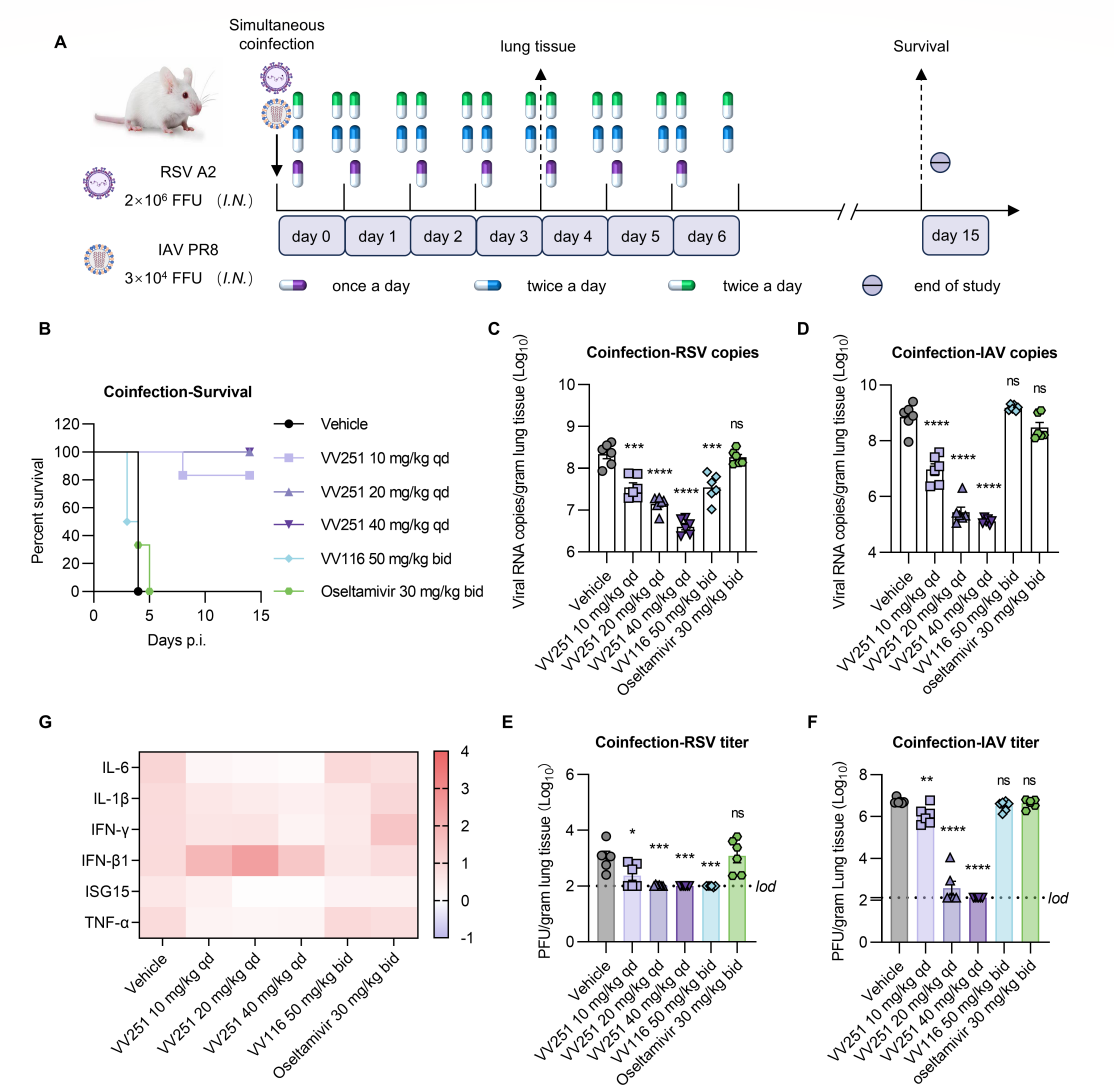
Efficacy of VV251 in RSV/IAV coinfection mouse model. (**A**) Schematic of the efficacy study in BALB/c mice. Two animal experiments were conducted: the first focuses on evaluating the effect of VV251 treatment on the mortality of mice, while the second aims to assess its impact on viral replication and lung pathology. Both experiments employed 6- to 8-week-old female BALB/c mice, which were intranasally coinfected with RSV and IAV simultaneously, followed by once daily oral administration of VV251. (**B**) Percentage of surviving vehicle-, VV251-, VV116-, and oseltamivir-treated mice after coinfection (*n* = 6). (**C** and **D**) Viral RNA levels in the lung tissue of vehicle-, VV251-, and ribavirin-treated mice on day 4 post-infection (*n* = 6). (**E** and **F**) Viral titers in lung tissue 4 days after infection (*n* = 6). (**G**) Cytokine gene expression was measured in the lungs on day 4 (*n* = 6). GAPDH was used as the internal reference gene.

Based on the broad-spectrum antiviral activity of VV251 against both RSV and IAV, along with the clinical reality of respiratory viral coinfections, we further evaluated the antiviral efficacy of VV251 in an RSV/IAV coinfection model. Considering that coinfections in humans typically manifest as sequential infections, we therefore initially developed a sequential RSV/IAV coinfection model in mice (Fig. S7). In this model, mice were infected with RSV followed by IAV challenge 24 h later, and the antiviral activity of VV251 was assessed. Oral administration of VV251 significantly reduced the viral loads of both RSV and IAV (Fig. S8). However, this sequential infection induced rapid body weight loss, reaching the predefined humane endpoint (≥20% wt loss) by day 2 post-coinfection, necessitating an adjustment of the viral challenge dose. Based on these observations and considering the multifactorial complexity of coinfection dynamics, we subsequently employed a simultaneous RSV/IAV coinfection model for the definitive antiviral evaluation of VV251, a model in which viral interference is minimized (33). This assessment was conducted in 6- to 8-week-old female BALB/c mice, with samples collected on day 4 post-infection. The BALB/c mouse model of RSV infection is nonlethal, whereas the BALB/c model of high-dose IAV PR8 (3 × 10^4^ focus forming units [FFU]) infection is lethal (32, 34). Hence, as shown in Fig. 5A, we investigated whether coinfection with RSV A2 and PR8 simultaneously would lead to mortality in BALB/c mice and whether this mortality could be alleviated by VV251 treatment.

**Fig 6:**
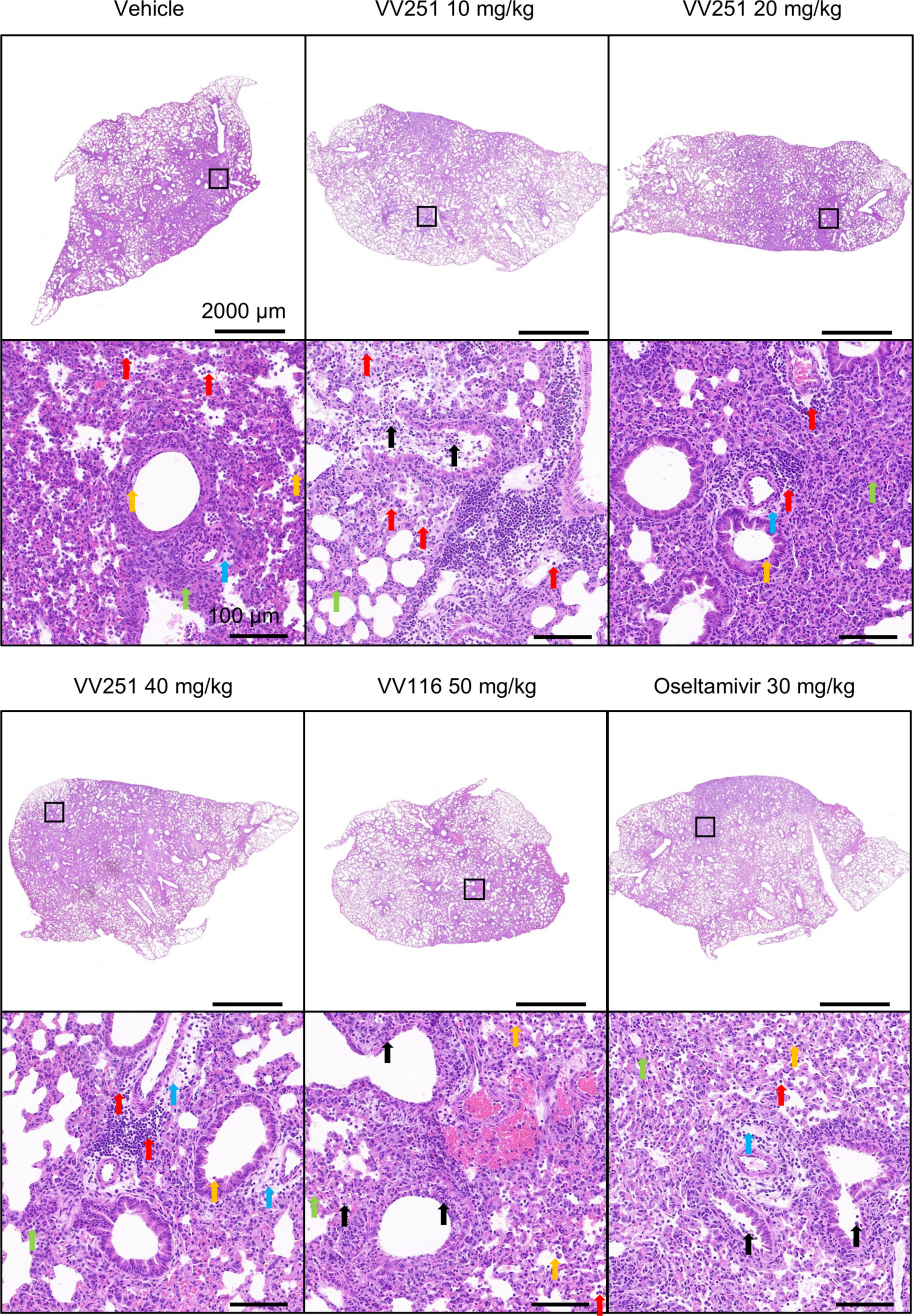
Histopathological analysis of the lung tissues from drug-treated mice compared with those of vehicle-treated mice. Red arrows indicate inflammatory cell infiltration, orange arrows indicate epithelial cell shedding, green arrows indicate thickening of the alveolar wall, blue arrows indicate thickening of perivascular connective tissue, and black arrows indicate cell necrosis, nuclear fragmentation, and condensation. Scale bars indicate 2,000 μm and 100 μm. In all panels, the symbols represent individual independent biological replicates, and the lines represent the mean values. Statistical significance compared to vehicle group was analysed by unpaired Student’s *t* test. *P < 0.05; **P < 0.01; ***P < 0.001; ****P < 0.0001; and ns, not significant.

In the RSV/IAV coinfection model, three doses were used to assess the antiviral activity of VV251. VV116 possesses anti-RSV activity but not anti-influenza activity, whereas the influenza inhibitor oseltamivir lacks reported anti-RSV effects (35–38). Therefore, we examined the antiviral activities of VV116 and oseltamivir as controls for monotherapy in addition to VV251. In contrast to the mice in the VV251 treatment groups, the mice in the vehicle and monotherapy groups died within 5 dpi (Fig. 5B). Although a mouse that received the lowest dose of VV251 (10 mg/kg) was euthanized because it reached the human endpoint of 20% weight loss at 8 dpi (Fig. 5B), it did not exhibit the ruffled fur phenomenon that is commonly observed after RSV A2 or PR8 infection. Unlike the VV251-treated group in the BALB/c mouse model of RSV A2 or PR8 monoinfection, lung damage (pathological score and histopathology) was not effectively alleviated in the BALB/c model infected with both RSV A2 and IAV PR8 simultaneously (Fig. 6; Fig. S11). Nevertheless, we observed an increase in body weight, as well as a reduction in the viral copy number and titer, in the VV251-treated groups compared with the vehicle-treated group (Fig. 5C through F; Fig. S9). We investigated the expression of inflammatory cytokines in the lungs to explore whether these compounds ameliorated lung damage by influencing the host immune response. Except for IFN-β1, the treatment with VV251 significantly reduced the levels of IL-6, IL-1β, and TNF-α compared to the vehicle control group and showed a tendency to decrease ISG15 and IFN-γ, although not significantly (Fig. 5G; Fig. S10). In summary, these results indicate that oral administration of VV251 once a day can effectively inhibit viral replication, reduce the expression of pro-inflammatory factors, and protect mice from death in the simultaneous RSV/IAV coinfection model.

## DISCUSSION

RSV and influenza virus are major causative agents of respiratory tract infections globally, leading to high morbidity and mortality rates every year (2, 4). However, several problems have been noted in the clinical treatment of both RSV infection and IAV infection. The clinical application of ribavirin in the treatment of RSV is limited due to its toxicity and efficacy, and the appearance of antiviral resistance in influenza virus is also a major concern. Therefore, developing new antiviral compounds that can effectively combat RSV and IAV is crucial. Previous studies have shown that 4′-FlU exhibits broad-spectrum antiviral activity against various RNA viruses, including SARS-CoV-2, RSV, influenza virus, chikungunya virus (CHIKV), enterovirus A71, and severe fever with thrombocytopenia syndrome virus (22, 23, 39–41). Studies concerning the mechanism of action (MOA) of 4′-FlU have revealed that 4′-FlU is a direct-acting antiviral agent (DAA) that targets viral RdRp and exerts antiviral activity through chain termination (22, 23). Compared to host-targeting antivirals, DAAs generally exhibit more effective activity and less toxicity, leading to the predominant use of DAAs for antiviral treatments (42). However, the poor chemical stability of 4′-FlU under water and phosphate buffer is likely to hinder further development. Previous studies have reported that after esterification, the chemical stability of derived 4′-FlU prodrugs is improved (24, 25). Based on the improvement strategy, we designed the compound VV251 that is a triester prodrug of 4′-FlU with high chemical stability under acidic (pH 1.5, simulating gastric fluid) and neutral (pH 6.8, simulating intestinal fluid) conditions. The stability in acidic to neutral environments greatly contributes to its drug-like properties. Moreover, following oral administration of equimolar doses in BALB/c mice, VV251 resulted in a significantly higher systemic exposure, as indicated by the larger AUC_0-*t*_, even though the prodrug VV251 and the parent nucleoside 4′-FlU exhibited comparable *T*_max_ values in mouse plasma.

In this study, we observed that VV251 exhibited antiviral activity against RSV and IAV *in vitro* and *in vivo*. Lieber et al. reported the antiviral activity of 4′-FlU against various RSV and IAV strains *in vitro*, with EC_50_ values ranging from nanomolar to low micromolar (22, 23). Additionally, in the RSV- or IAV-infected mouse model, oral administration of 4′-FlU was efficacious in reducing the viral load of RSV or IAV. Despite the subtle difference in the virus strain and methods, our research results coincided with the previously reported antiviral activity of 4′-FlU, which indicates that our experimental setup is workable and tenable. Further evaluation of VV251 against DNA viruses (HSV-1 and VZV) *in vitro* revealed minimal antiviral activity (Fig. S2).

Owing to the frequent occurrence of respiratory virus coinfections and considering the substantial antiviral activity of VV251 against both RSV and IAV, we established a mouse model of RSV/IAV coinfection and observed that VV251 exhibited antiviral activity against RSV A2 and PR8 in a simultaneously coinfected mouse model. It has been reported that coinfection of RSV and IAV can alter the replication of both viruses, which is influenced by the sequence of infection and the temporal interval between the two infections (18, 43–45). Additionally, compared with the groups infected with RSV and IAV sequentially, when RSV and IAV were coinfected simultaneously, a significant increase in the viral load was observed at 2 dpi, and this viral load was also higher than that in the case of single virus infection (46). However, the RSV load decreased rapidly at 7 dpi (46). Here, we coinfected mice with RSV and IAV simultaneously to establish an animal simultaneous RSV/IAV coinfection model and sacrificed them at 4 dpi to detect the antiviral activity of VV251. In this coinfection model, mice that were coinfected with RSV/IAV simultaneously presented disease symptoms, including weight loss and ruffled fur. Moreover, as documented in previous studies, high RSV and IAV viral loads were detected simultaneously in the lung tissues of the mice. After the oral administration of VV251 once daily, a significant reduction was observed not only in the mortality of the mice but also in the viral loads of RSV and IAV in the mouse RSV/IAV coinfection model. The reduction in the RSV and IAV viral loads in the lung tissue of the mice ranged from 0.6 to 4.6log_10_ at 4 dpi. The attenuation of lung damage and the modulation of inflammatory factor levels were inferior to those in mice with single infections with RSV or IAV. This difference might be attributable to coinfection with RSV and IAV, which exacerbates disease severity and prolongs the convalescence period.

Although we have shown that VV251 exhibits highly effective broad-spectrum activity against respiratory virus infection in cell culture and animal models, some limitations remain. First, the MOA of VV251 has not been fully elucidated. Even though the antiviral mechanism of 4′-FlU targeting RdRp has been reported, the potential off-target effects of VV251 and its metabolites cannot be excluded. Off-target toxicity from poor polymerase selectivity is a key hurdle for broad-spectrum nucleoside analogs, causing mitochondrial toxicity (via Pol γ) and host nucleic acid disruption, which can cause cytotoxicity, mutagenesis, or immunosuppression (21). Thus, a high SI of a nucleoside analog is necessary. Additionally, defining the therapeutic window requires comprehensive preclinical profiling for mitochondrial dysfunction and genomic instability. Second, the resistance characteristics of VV251 need to be further investigated. Due to 4′-FlU showing high resistance barrier to influenza viruses, enteroviruses, and CHIKV (40, 47, 48), we hypothesize that VV251, the prodrug of 4′-FlU, might possess a higher resistance barrier as well. Additionally, combination therapy targeting multiple targets should be further investigated for more effective antiviral strategies and block of resistance escape as conducted in anti-HIV, -HCV, and -SARS-CoV-2 (49–51). Finally, our investigations were confined to a mouse model and lacked validation in larger or more diverse animal models. Furthermore, our evaluation model requires further expansion: (i) Although the therapeutic window remains to be systematically assessed to determine its impact on VV251’s antiviral activity in mono- and coinfection settings, previous studies have demonstrated that 4′-FlU, the parent compound, retains significant antiviral activity against both RSV and IAV when administered orally at 24, 48, and 72 h post-infection (22, 23). Given that VV251 is a prodrug that releases 4′-FlU *in vivo*, it is reasonable to infer that VV251 would also effectively suppress viral replication. The absence of this systematic evaluation constitutes a limitation of the present study; (ii) Direct head-to-head comparison in the RSV-infected BALB/c mouse model revealed no significant difference in antiviral efficacy between VV251 and 4′-FlU, which aligns with their similar pharmacokinetic profiles in mice (Fig. S12). Critically, VV251 demonstrated a significantly enhanced PK profile in non-human primates (52), supporting its potential as an improved clinical candidate. However, this head-to-head evaluation was not extended to the IAV mono-infection or RSV/IAV coinfection models, which represents a limitation of the present study; (iii) Given the complexity of coinfections, which can be influenced by factors such as virus strain, dose, infection sequence, and timing, we have only evaluated the antiviral activity of VV251 in a sequential coinfection mouse model (with a 24 h interval between RSV and IAV infections) and a simultaneous coinfection model. The effects of varying infection intervals (>24 h), virus strains, and doses have yet to be explored. Given that the current findings are based on preclinical studies, the clinical efficacy of VV251 remains uncertain and warrants further investigation.

### Conclusion

In conclusion, our results suggest that VV251 is a broad-spectrum antiviral nucleoside analog against respiratory viruses. Based on the reported broad-spectrum antiviral activity of 4′-FlU against enteroviruses, alphaviruses, bunyaviruses, arenaviruses, etc. (26, 39–41), we speculate that VV251, a prodrug of 4′-FlU, has potential antiviral activity against these viruses. Furthermore, this study indicates that VV251 has promising effects on emerging pathogens and viral coinfections. Given the potent and broad-spectrum antiviral activity of VV251, further ongoing exploration of the application value of VV251 is important.

## MATERIALS AND METHODS

### Animals

All the *in vivo* experiments of RSV and simultaneous RSV/IAV coinfection were performed in 6- to 8-week-old female BALB/c mice (22, 45, 46). Male BALB/c mice aged 3–4 weeks were used for IAV *in vivo* experiments (31, 53). Male BALB/c mice aged 6–8 weeks were used for pharmacokinetic studies.

### Cells, viruses, and antiviral compounds

HEp-2 (ATCC CCL-23), Vero E6 (ATCC-1586), A549 (ATCC CCL-185), MDCK (ATCC CCL-34), Vero (ATCC CCL-81), and ARPE (ATCC CRL-2302) cells were cultured in Dulbecco’s modified Eagle’s medium (DMEM) supplemented with 10% fetal bovine serum (FBS) at 37°C in a humidified atmosphere with 5% CO_2_. 16HBE cells, purchased from Merck Millipore, were maintained in our laboratory in RPMI 1640 (Gibco) supplemented with 10% FBS. RSV was propagated on HEp-2 cells in DMEM supplemented with 2% FBS at 37°C. The RSV ON1 strain was isolated from nasopharyngeal swab samples collected clinically and was used after single plaque purification, with the assurance that the number of passages was within 10 to maintain low passage numbers. Influenza virus strain A/Puerto Rico/8/1934(H1N1) was propagated in MDCK cells in DMEM containing 0.1% bovine serum albumin (BSA) and 1 μg/mL TPCK-trypsin. Virus stock titers were determined by immuno-plaque assays, and stocks were stored in aliquots at −80°C. Virus stocks (HSV-1 in Vero cells and VZV in ARPE-19 cells) were harvested at 72 h post-infection for titer determination via plaque assay.

VV251, 4′-FlU, and VV116 were obtained from the Shanghai Institute of Materia Medica, Chinese Academy of Sciences. Ribavirin, favipiravir, and oseltamivir were purchased from MedChemExpress. All the compounds were initially dissolved in dimethyl sulfoxide (DMSO) for *in vitro* studies and subsequently diluted in maintenance medium. For *in vivo* administration via oral gavage, the compounds were formulated in a solution of 5% DMSO, 5% ethanol, 40% PEG400, and 50% saline.

### Solution stability studies

A stability study of 4′-FlU and its prodrug VV251 was conducted in buffers of different pH values. The pH 1.5 buffer was prepared by adding 3.73 mL concentrated hydrochloric acid to 1 L of deionized water. The pH 6.8 buffer was prepared by mixing 250 mL of 0.2 mol/L potassium dihydrogen phosphate solution with 112.0 mL of 0.2 mol/L sodium hydroxide solution, followed by dilution to a final volume of 1,000 mL with deionized water. For each compound, 1 mg was dissolved in a mixture consisting of 1 mL of the corresponding buffer (pH 1.5 or pH 6.8) and 1 mL of DMSO. The purity of the solution was analyzed by HPLC at 0, 4, 12, and 24 h. The purity changes at each time point (4, 12, and 24 h) were calculated by subtracting the initial purity (at 0 h) from the purity measured at the respective time point.

### Pharmacokinetic studies

The pharmacokinetic studies were performed at Suzhou Fangkun Medical Technology Co., Ltd. The dosing vehicles used were 5% DMSO, 5% ethanol, 40% PEG 400, and 50% saline. Multiple plasma samples were collected from the orbital veins of the three mice in each group at 5 min, 0.25, 0.5, 1.0, 2.0, 4.0, 6.0, 8.0 h, and 24 h postdosing. The samples were transferred to EDTA-K2 tubes and then centrifuged at 11,000 rpm for 5 min. The plasma samples were aliquoted and frozen at −70°C for testing. All these operations were conducted in an ice water bath. The plasma concentrations of 4′-FlU were determined using a liquid chromatography-tandem mass spectrometry system.

### Evaluation of the antiviral efficacy of the compounds *in vitro*

Subconfluent HEp-2 cells were inoculated with RSV A2 or RSV B at a multiplicity of infection (MOI) of 0.01 following a 1 h incubation in medium containing serially diluted compounds. After 2 h of infection at 37°C, the mixture was removed and replaced with fresh medium containing the corresponding compound. At 48 hpi, HEp-2 cells were harvested using RNAiso Plus (TaKaRa). Viral RNA was then extracted and quantified by real-time fluorescence quantitative PCR (qRT-PCR) with TB Green Premix Ex Taq II (Tli RNaseH Plus; TaKaRa) and evaluated via the relative quantification method. For the dose‒response assays conducted using A549 cells and 16HBE cells, the procedures were largely consistent with those described above. However, notably, the MOIs differed. For A549 cells, the MOI was set at 0.05, whereas for 16 HBE cells, the MOI was 0.01. The clinical strain RSV ON1 was used to infect HEp-2 cells at an MOI of 0.01, and the infection process was maintained for a total of 72 h. The primers used for qRT-PCR were provided in the supporting information.

Similarly, subconfluent MDCK cells cultured in 48-well plates were infected with PR8 at an MOI of 0.01 following an incubation in media containing the compounds. The cells were subsequently incubated at 37°C in an atmosphere with 5% CO_2_ for 1 h. Then, the inoculum was removed, and various concentrations of the test compounds, which had been diluted in maintenance medium, were added to the cells. After 48 hpi, the viral RNA in the supernatant was extracted using the VAMNE Virus DNA/RNA Extraction Kit 3.0 (Vazyme), and its quantity was determined by qRT-PCR. The viral copy number was estimated through the standard curve method. The primers used for qRT-PCR were provided in the supporting information. These experiments were independently performed three times.

Antiviral assays against HSV-1 and VZV were conducted in Vero and ARPE cells, respectively. Prior to the assay, cells were seeded into 48-well plates. Cells were then incubated with serial dilutions of VV251 or 4′-FlU for 1 h at 37°C. Subsequently, they were infected with HSV-1 (MOI of 0.2) or VZV (MOI of 0.2). After 1 h of viral adsorption, the inoculum was removed from Vero cells and replaced with fresh DMEM containing 2% FBS and the corresponding concentration of the compound, which was maintained for the duration of the experiment. Supernatants were harvested at 48 hpi for viral load determination. For VZV-infected ARPE cells, the supernatant was not replaced. At 48 hpi, the entire culture plate was frozen at −80°C. The supernatant was collected after thawing for subsequent viral titer analysis. The primers used for qRT-PCR were provided in the supporting information.

### Cytotoxicity assays

Antiviral compounds were serially diluted in twofold increments from a 1,000 μM stock solution in maintenance medium and then plated on 96-well microplates in triplicate. The cells within the plates were subsequently cultured for 48 h. Afterward, Cell Counting Kit-8 reagent (Beyotime) was applied to the cells, which were subsequently incubated for 1–2 h. Cell viability was determined by measuring the absorbance at 450 nm. The cells in the control wells were treated with maintenance medium supplemented with 0.5% DMSO. The CC_50_ values were determined using GraphPad Prism (version 8.0) with a variable slope (four parameters). These experiments were independently replicated three times.

### VV251 anti-RSV A2 efficacy studies *in vivo*

To evaluate the efficacy of VV251, the mice in the VV251 groups (20 mg/kg, 40 mg/kg, and 60 mg/kg) were administered the compound via oral gavage once daily (qd, 24 h interval) for 4 consecutive days after intranasal inoculation with RSV A2 (4 × 10^6^ FFU) 1‒2 h. The mice were anesthetized with isoflurane. The ribavirin group (50 mg/kg), which served as a positive control, was treated using the same protocol except that the drug was administered bid (12 h interval). The mice were monitored daily for weight changes. They were sacrificed 4 days after infection, and the lungs were then harvested. The right lungs were weighed and homogenized in 400 μL of PBS to determine the lung viral titers, viral loads, and inflammatory factor levels. The left lungs were fixed with 4% paraformaldehyde for hematoxylin-eosin staining and histological analyses. Total RNA was extracted from the lung homogenates using the MiniBEST Viral RNA/DNA Extraction Kit Ver.5.0 (TaKaRa), and the levels of viral RNA were quantified using TB Green Premix Ex Taq II (Tli RNaseH Plus). The viral RNA copy number was calculated based on the concentration of standard plasmids for the RSV A2 F protein. The primers used for the qRT-PCR analysis of host cytokine mRNA expression were provided in the supporting information.

### PR8 lethal mouse model

For the efficacy studies, mice were randomly divided into five groups (*n* = 7), and then isoflurane-anesthetized mice were infected intranasally with 3 × 10^4^ FFU of PR8. Treatments (vehicle, VV251 10 mg/kg, VV251 20 mg/kg, VV251 30 mg/kg, or favipiravir 200 mg/kg) were administered at 1 h post-infection by oral gavage and continued once daily (qd, 24 h interval) for a duration of 4 days. The mice were monitored daily for weight changes. At 4 days post-infection, the lung tissue was harvested for virus titration or histopathological assessments, using the same methods as above. The primers used for qRT-PCR to measure host cytokine mRNA expression were provided in the supporting information.

### Inhibitory effects of VV251 on RSV A2 and PR8 coinfection *in vivo*

For the establishment of the coinfection mouse model, animals were divided into the following groups: uninfected (PBS), single-infected (RSV or IAV), and sequential co-infected (RSV followed by IAV, or IAV followed by RSV, with a 24 h interval between infections). The mice were anesthetized with isoflurane and then challenged with RSV A2 (4 × 10⁶ FFU per mouse) or IAV PR8 (3 × 10⁴ FFU per mouse). The body weight of the mice was measured daily, and lung tissues were obtained on 2 dpi and 3 dpi. The detection method was the same as above.

To evaluate the antiviral activity of VV251 under sequential RSV-then-IAV coinfection, mice were anesthetized with isoflurane and inoculated intranasally with RSV A2 at a dose of 4 × 10⁶ FFU per mouse. Twenty-four hours later, the mice were challenged with IAV PR8 at a dose of 3 × 10⁴ FFU per mouse. For antiviral treatment, VV251 (10, 20, or 40 mg/kg) was administered orally once daily (q.d., 24 h intervals) beginning 1–2 h post-coinfection and continued for 2 consecutive days. As positive controls, VV116 (25 mg/kg) and oseltamivir (20 mg/kg) were administered orally bid (12 h intervals), also starting 1–2 h post-coinfection. All mice were euthanized on day 2 post-coinfection for viral load analysis, which was performed using the same methods as previously described.

The mice were anesthetized with isoflurane and simultaneously inoculated with RSV A2 (2 × 10^6^ FFU) and PR8 (3 × 10^4^ FFU) intranasally. For antiviral treatment, 10, 20, or 40 mg/kg VV251 was administered orally once daily (qd, 24 h interval) starting at 1‒2 h after infection. For the evaluation of VV251 efficacy, all the compounds were administered continuously for 4 days, while the mice were continuously treated for 7 days until the survival rate was determined. Moreover, VV116 (50 mg/kg) and oseltamivir (30 mg/kg) were administered orally 1‒2 h after infection bid (12 h interval). Changes in the body weights and survival rates of the mice were recorded daily for up to 15 days after infection. Mice showing >20% wt loss were humanely euthanized according to humane endpoints. The mice were euthanized for an examination of the viral load and lung tissue damage in the lungs at 4 days post-infection. The detection method was the same as above.

### Virus titration from lung tissue samples

For the measurement of viral titers in the tissues of infected animals, immuno-plaque assays were performed. Lung homogenates were clarified by centrifugation at 5,000 rpm for 10 min, and 10-fold serial dilutions of tissue homogenates were prepared in serum-free medium. The dilutions were added to Vero E6 cells in duplicate, replaced with 1% methylcellulose containing 2% FBS after an incubation for 2 h, and fixed with 4% formaldehyde after 5 days. The cells were permeabilized with a solution containing 0.3% Triton X-100 and 5% skim milk for 30 min and stained with an anti-RSV goat polyclonal antibody (Abcam) overnight at 4°C. HRP-conjugated donkey anti-goat IgG (H + L) (ABclonal) was used as the secondary antibody, and the sections were then stained with a DAB staining kit (TIANGEN). MDCK cells and 1% methylcellulose supplemented with 0.1% BSA and 1 μg/mL TPCK-trypsin were used to determine the PR8 viral titer. An influenza virus nucleoprotein antibody (Genetex) and HRP-conjugated AffiniPure goat anti-rabbit IgG (H + L) (Proteintech) were used as the primary and secondary antibodies, respectively.

### Statistical analysis

All statistical analyses were performed using GraphPad Prism software (version 8.0). Outliers in the expression levels of inflammatory factors were identified and excluded using the ROUT method, with *Q* set to 1%. Differences between groups were assessed for statistical significance using an unpaired Student’s *t*-test or one-way analysis of variance. *P < 0.05; **P < 0.01; ***P < 0.001; ****P < 0.0001; and ns, not significant.

## Data Availability

The data that support the findings of this study are available from the corresponding authors upon request.

## SUPPLEMENTAL MATERIAL

Supplemental material jvi.00006-26-s0001.docx Tables S1 to S4; Fig. S1 to S12.

ASM does not own the copyrights to Supplemental Material that may be linked to, or accessed through, an article. The authors have granted ASM a non-exclusive, world-wide license to publish the Supplemental Material files. Please contact the corresponding author directly for reuse.
