# Supplementary material for: The oral nucleoside analogue inhibitor VV251 effectively inhibits coinfection by respiratory syncytial virus and influenza A virus
Source: J Virol. 2026 Apr 21;100(5):e00006-26. doi: 10.1128/jvi.00006-26 (PMC13185581; doi:10.1128/jvi.00006-26)
Supplement: Supplemental material — Tables S1 to S4; Fig. S1 to S12. [file jvi.00006-26-s0001.docx]

**Supporting information**

**Table S1** Purity changes of 4′-FlU and VV251 in pH 1.5 and pH 6.8 buffers over 24 h

| Conditions | Time (h) | Change of purity (%) in 24 hours | | | |
| --- | --- | --- | --- | --- | --- |
|  |  | **4′-FlU** | | **VV251** | |
|  |  | **Purity (%)** | **Deviation (%)** | **Purity (%)** | **Deviation (%)** |
| pH 1.5 | **0** | 94.70 | / | 98.92 | / |
|  | **4** | 67.34 | -27.36 | 94.56 | -4.36 |
|  | **12** | 36.99 | -57.71 | 87.68 | -11.24 |
|  | **24** | 2.46 | -92.24 | 70.07 | -28.85 |
| pH 6.8 | **0** | 96.44 | / | 97.54 | / |
|  | **4** | 80.86 | -15.58 | 95.83 | -1.71 |
|  | **12** | 60.17 | -36.27 | 90.35 | -7.19 |
|  | **24** | 19.97 | -76.47 | 78.15 | -19.39 |

**Table S2** Concentrations of 4′-FlU in BALB/c plasma after oral administration of 4′-FlU (10 mg/kg) or VV251 (20 mg/kg) (n=3 mice per group).

| Time  (h) | **4′-FlU 10 mg/kg PO** | | | | | **VV251 20 mg/kg PO** | | | | |
| --- | --- | --- | --- | --- | --- | --- | --- | --- | --- | --- |
|  | **1** | **2** | **3** | **Mean** | **SD** | **7** | **8** | **9** | **Mean** | **SD** |
|  | **Concentration (ng/mL)** | | | | | **Concentration (ng/mL)** | | | | |
| 0.083 | 2820 | 2610 | 2820 | 2750 | 121 | 3630 | 7330 | 3540 | 4833 | 2163 |
| 0.25 | 8660 | 7190 | 9820 | 8557 | 1318 | 6970 | 11500 | 5360 | 7943 | 3184 |
| 0.50 | 7530 | 3960 | 6730 | 6073 | 1873 | 6190 | 6390 | 5470 | 6017 | 484 |
| 1.00 | 2970 | 2420 | 4750 | 3380 | 1218 | 4850 | 5240 | 4230 | 4773 | 509 |
| 2.00 | 1010 | 2090 | 2010 | 1703 | 602 | 3080 | 2070 | 3990 | 3047 | 960 |
| 4.00 | 724 | 742 | 1450 | 972 | 414 | 2050 | 1060 | 2100 | 1737 | 587 |
| 6.00 | 288 | 507 | 957 | 584 | 341 | 880 | 1020 | 930 | 943 | 71 |
| 8.00 | 237 | 476 | 851 | 521 | 310 | 435 | 686 | 876 | 666 | 221 |
| 24.00 | 205 | 332 | 361 | 299 | 83 | 244 | 211 | 217 | 224 | 18 |

**Table S3** PK parameters for 4′-FlU in mice. Calculation of PK parameters for 4′-FlU following oral administration of 4′-FlU (10 mg/kg) and VV251 (20 mg/kg) in BALB/c mice (n=3 mice per group).

| Compound | **Route** | **T_1/2_**  **(h)** | **T_max_**  **(h)** | **C_max_**  **(ng/mL)** | **AUC_0-t_**  **(ng·h/mL)** | **AUC_0-∞_**  **(ng·h/mL)** | **MRT_0-∞_**  **(h)** |
| --- | --- | --- | --- | --- | --- | --- | --- |
| 4′-FlU | PO | 19.15 | 0.25 | 8556.67 | 19693.98 | 27965.32 | 19.53 |
| VV251 |  | 9.20 | 0.25 | 7943.33 | 25809.60 | 28782.46 | 8.38 |

**Table S4** Forward and reverse primer sequences for the detection of qRT-PCR in this study

|  | **FORWARD PRIMER SEQUENCE** | **REVERSE PRIMER SEQUENCE** |
| --- | --- | --- |
| **RSV A-F** | 5’-CGAGCCAGAAGAGAACTACCA-3’ | 5’-CCTTCTAGGTGCAGGACCTTA-3’ |
| **RSV B-N** | 5’-TGACACTCCCAATTATGATGTGC-3’ | 5’-GAATCATGCCTATATTCTGGAGCC-3’ |
| **Human GAPDH** | 5’-GAAGATGGTGATGGGATTTC-3’ | 5’-GAAGGTGAAGGTCGGAGTC-3’ |
| **IAV PR8-HA** | 5’-CTGCTCGAAGACAGCCACAAC-3’ | 5’-GAAGCAGTGGGTCGCATTCT-3’ |
| **Mouse IL-6** | 5’-TCTATACCACTTCACAAGTCGGA-3’ | 5’-GAATTGCCATTGCACAACTCTTT-3’ |
| **Mouse IL-8** | 5’-TGTTGAGCATGAAAAGCCTCTAT-3’ | 5’-AGGTCTCCCGAATTGGAAAGG-3’ |
| **Mouse IFN-β1** | 5’-AGCTCCAAGAAAGGACGAACA-3’ | 5’-GCCCTGTAGGTGAGGTTGAT-3’ |
| **Mouse ISG15** | 5’-AGTGATGCTAGTGGTACAGAACT-3’ | 5’-CAGTCTGCGTCAGAAAGACCT-3’ |
| **Mouse TNF-α** | 5’-ATCGGTCCCCAAAGGGATGA-3’ | 5’-GCTCCTCCACTTGGTGGTTT-3’ |
| **Mouse IL-1β** | 5’-TTGACGGACCCCAAAAGATG-3’ | 5’-AGAAGGTGCTCATGTCCTCA-3’ |
| **Mouse IL-4** | 5’-GGTCTCAACCCCCAGCTAGT-3’ | 5’-GCCGATGATCTCTCTCAAGTGAT-3’ |
| **Mouse IFN-γ** | 5’-ATGAACGCTACACACTGCATC-3’ | 5’-CCATCCTTTTGCCAGTTCCTC-3’ |
| **Mouse GAPDH**  **HSV-1**  **VZV** | 5’-TGGTGAAGGTCGGTGTGAAC-3’  5’-CCGAGCCGATGACTTACTG-3’  5’-GTGCCAATAGAACAATAACAACCA-3’ | 5’-GAAGGGGTCGTTGATGGCAA-3’  5’-CCGATATGAGGAGCCAGAAC-3’  5’-ACCACGACGAGGAGATACG-3’ |


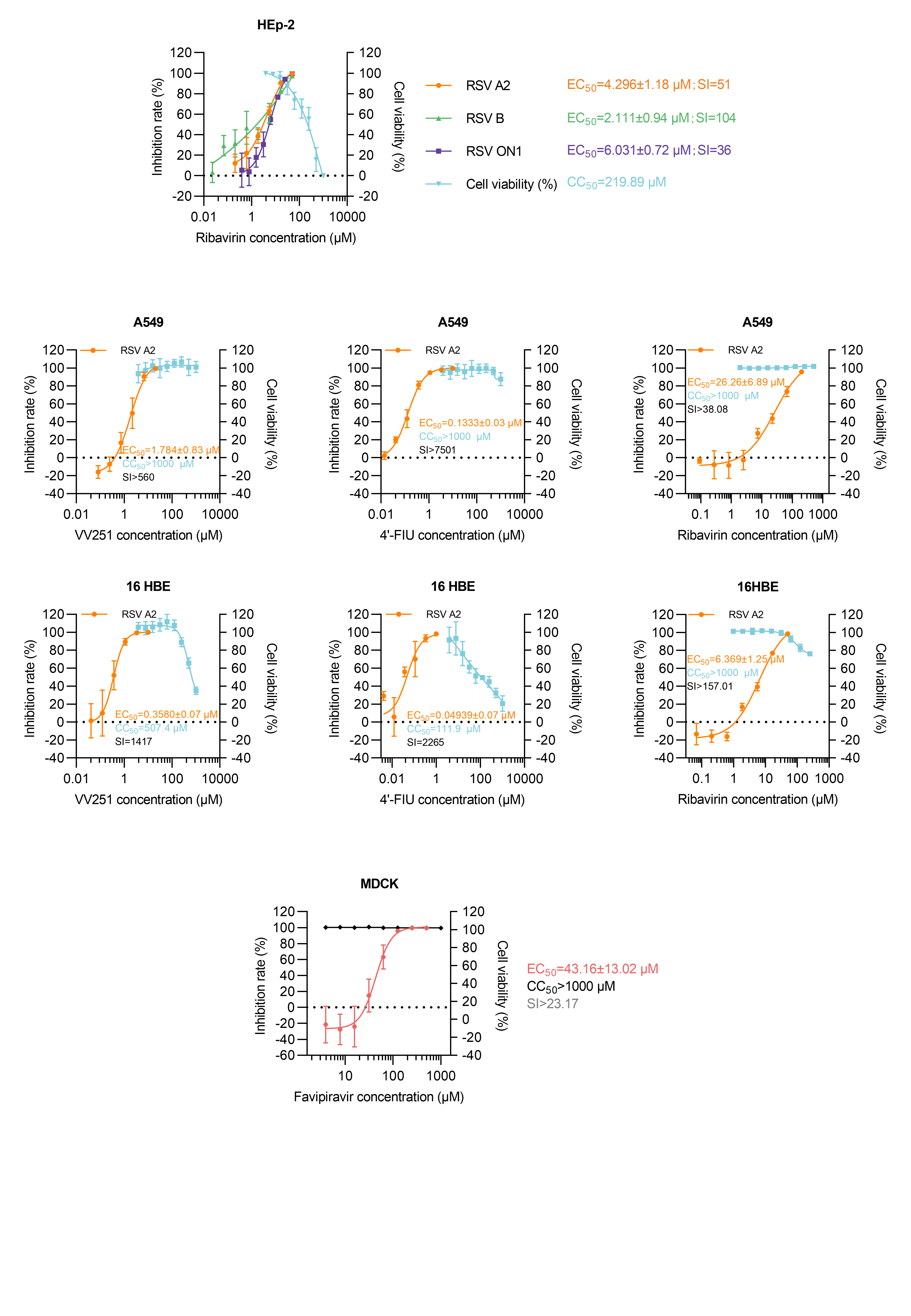


**FIG S1** VV251 effectively inhibits RSV A2 replication in A549 and 16 HBE cells. Viral inhibition was determined by real-time fluorescence quantitative PCR. The EC_50_ values and CC_50_ values were calculated using a 4-parameter variable slope regression model. The symbols represent independent repeats (n=3).


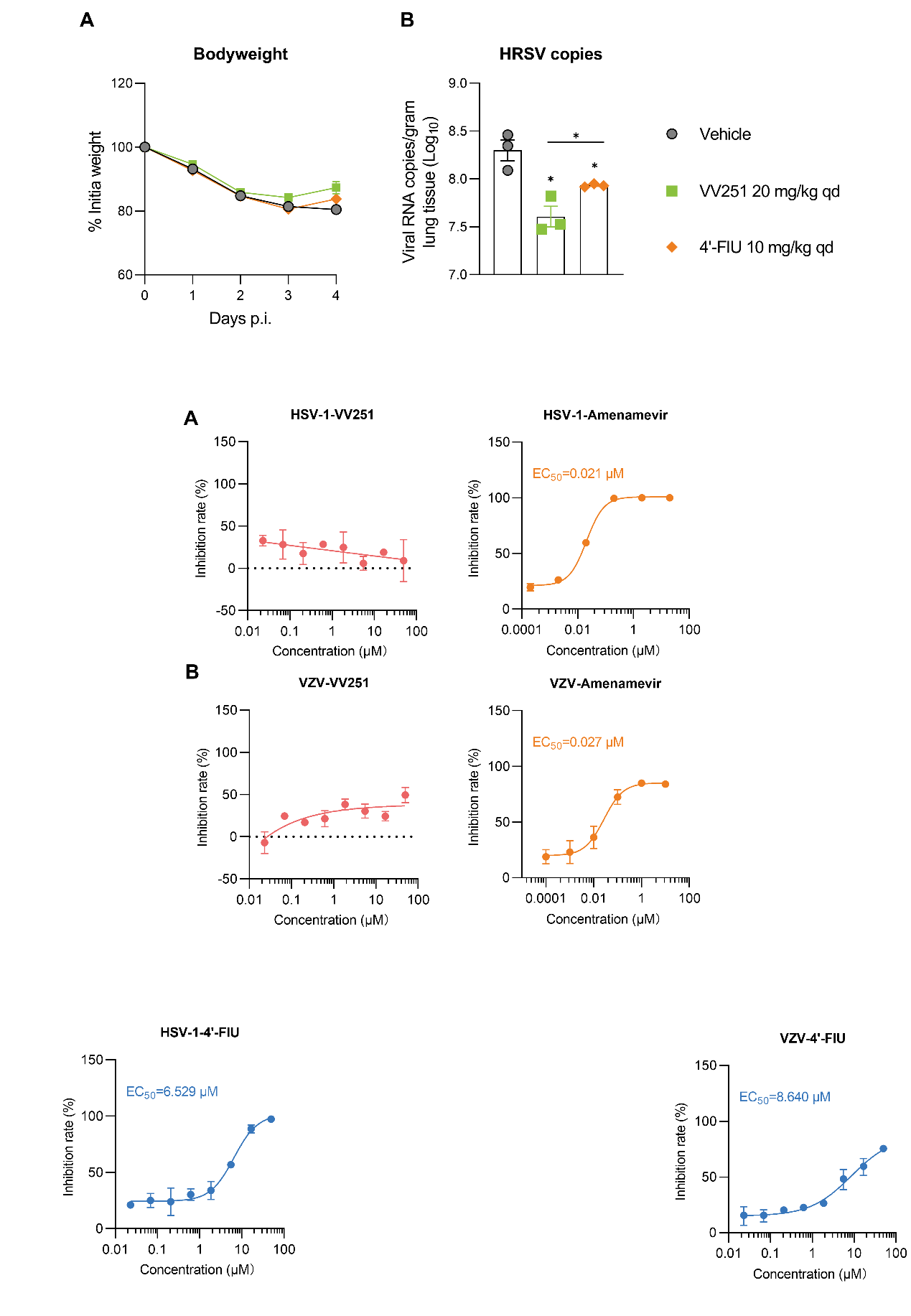


**FIG S2** Antiviral activities of VV251 against HSV-1 and VZV. Viral inhibition was determined by real-time fluorescence quantitative PCR. The EC_50_ values and CC_50_ values were calculated using a 4-parameter variable slope regression model. The data are presented as the mean values of independent experiments (n=2) ± standard deviation (SD).


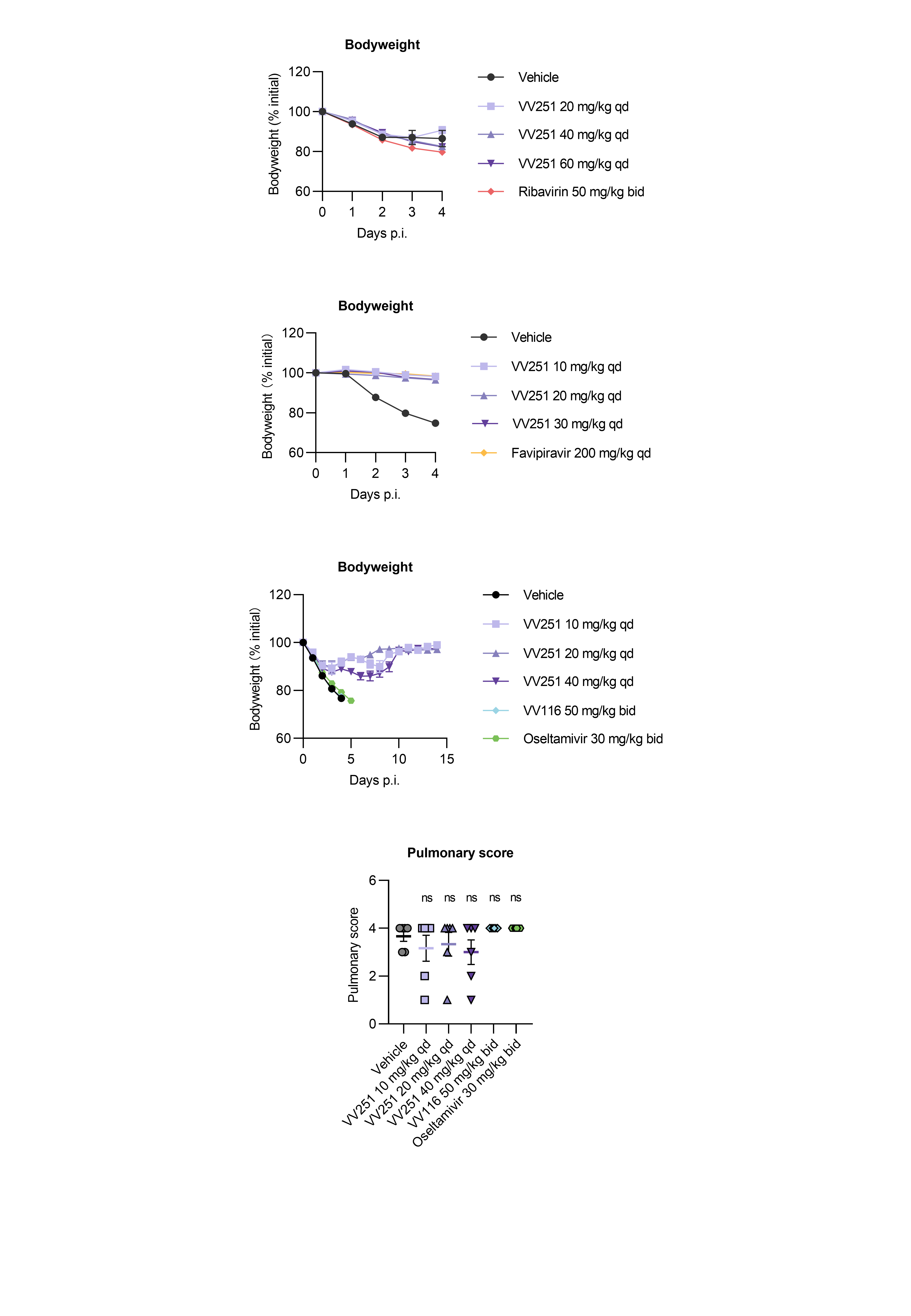


**FIG S3** Shown is the percentage change in body weight compared with the starting body weight of BALB/c mice infected with RSV A2 (n=6). The symbols represent the means ± SEM.


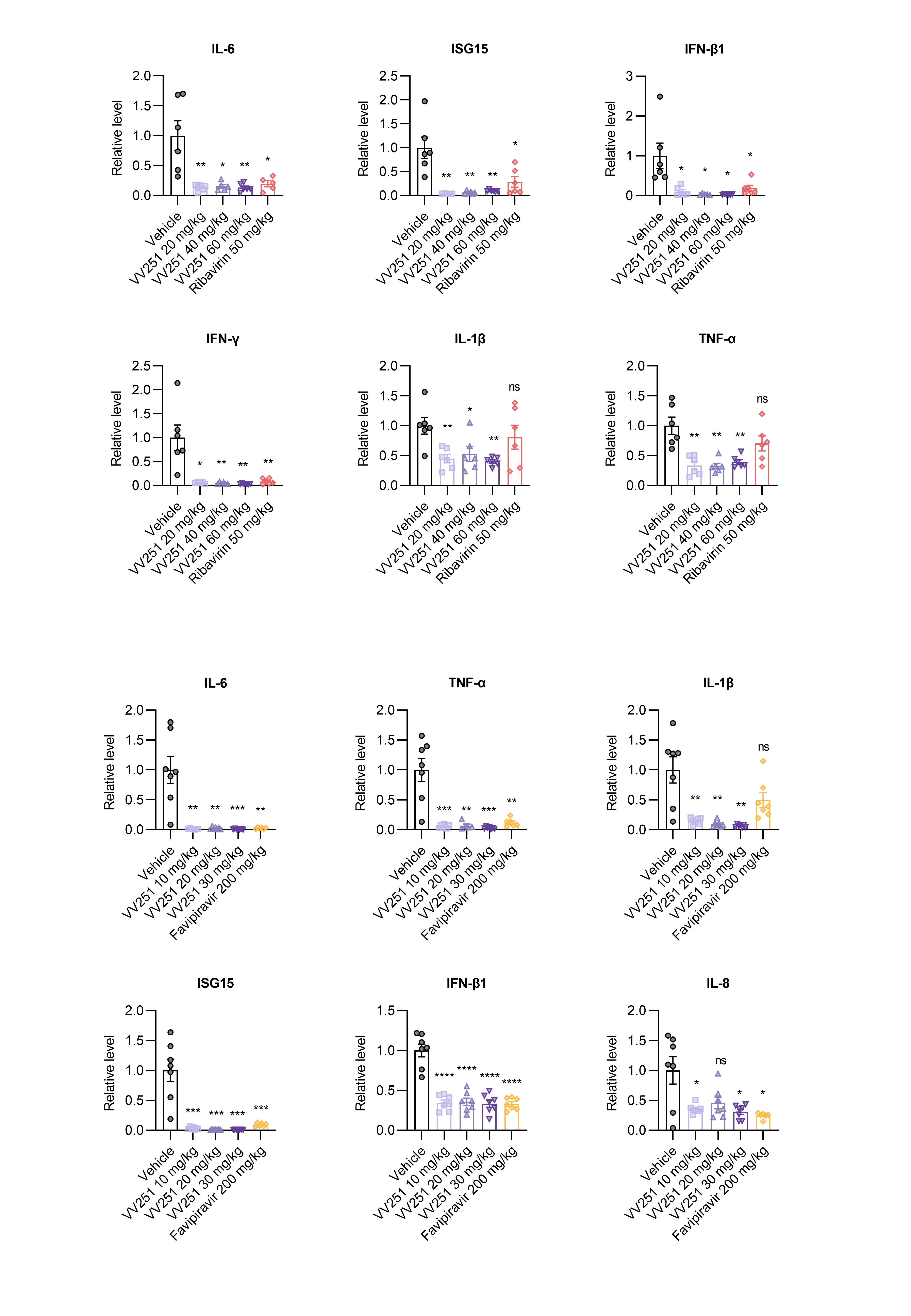


**FIG S4** Cytokine gene expression was measured in the lungs of mice infected with RSV on day 4 (n=6). GAPDH gene was used as the internal reference gene. The symbols represent individual values, and the error bars indicate the SEM. The ROUT method in GraphPad was used to remove the outliers from the data, and Q was set to 1%. Statistical significance compared to vehicle group was analysed by unpaired Student’s t test. **P* < 0.05; ***P* < 0.01; ****P* < 0.001; *****P* < 0.0001; and ns, not significant.


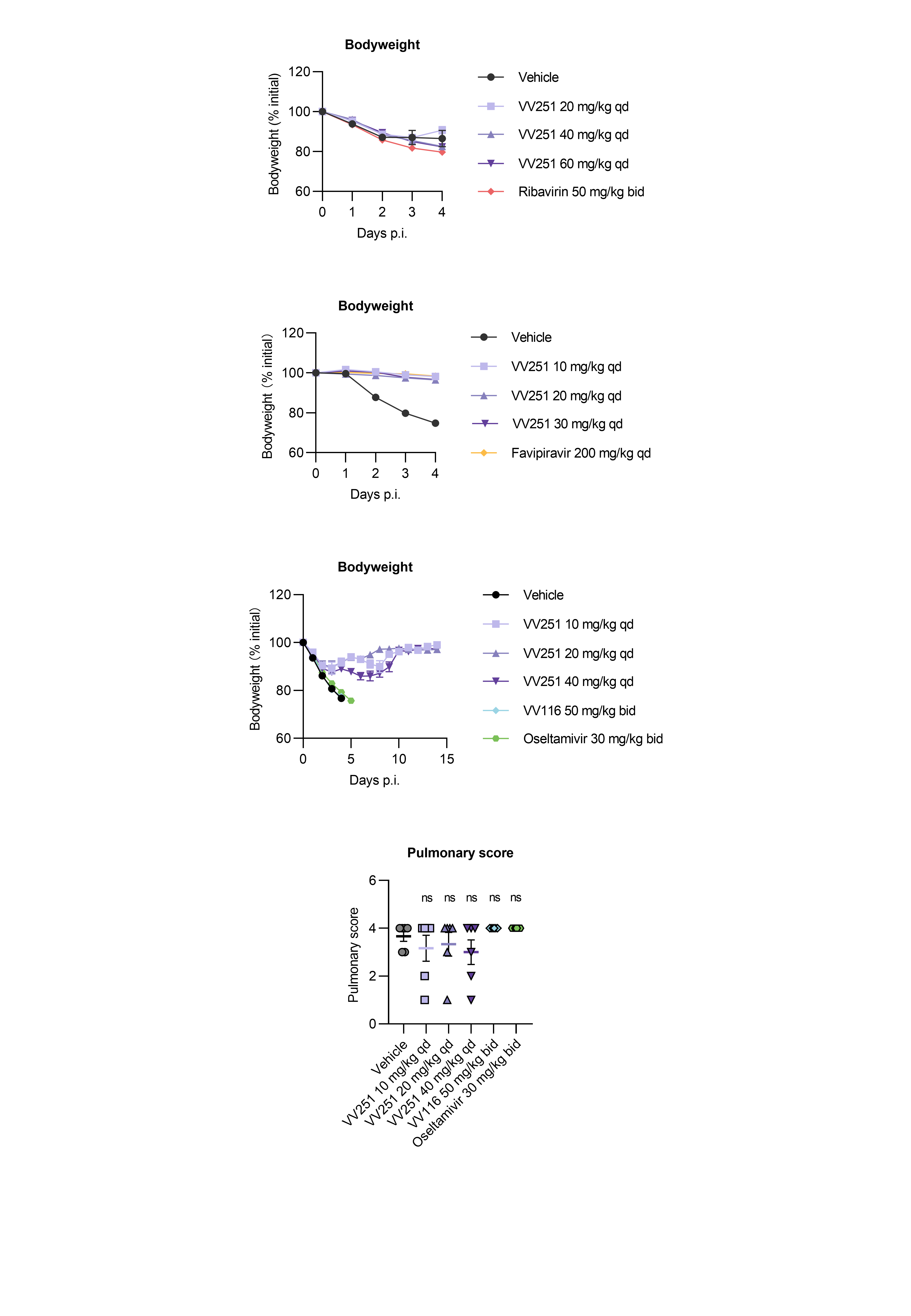


**FIG S5** Body weight changes in BALB/c mice challenged with IAV PR8 (n=7). The symbols represent the means ± SEM.


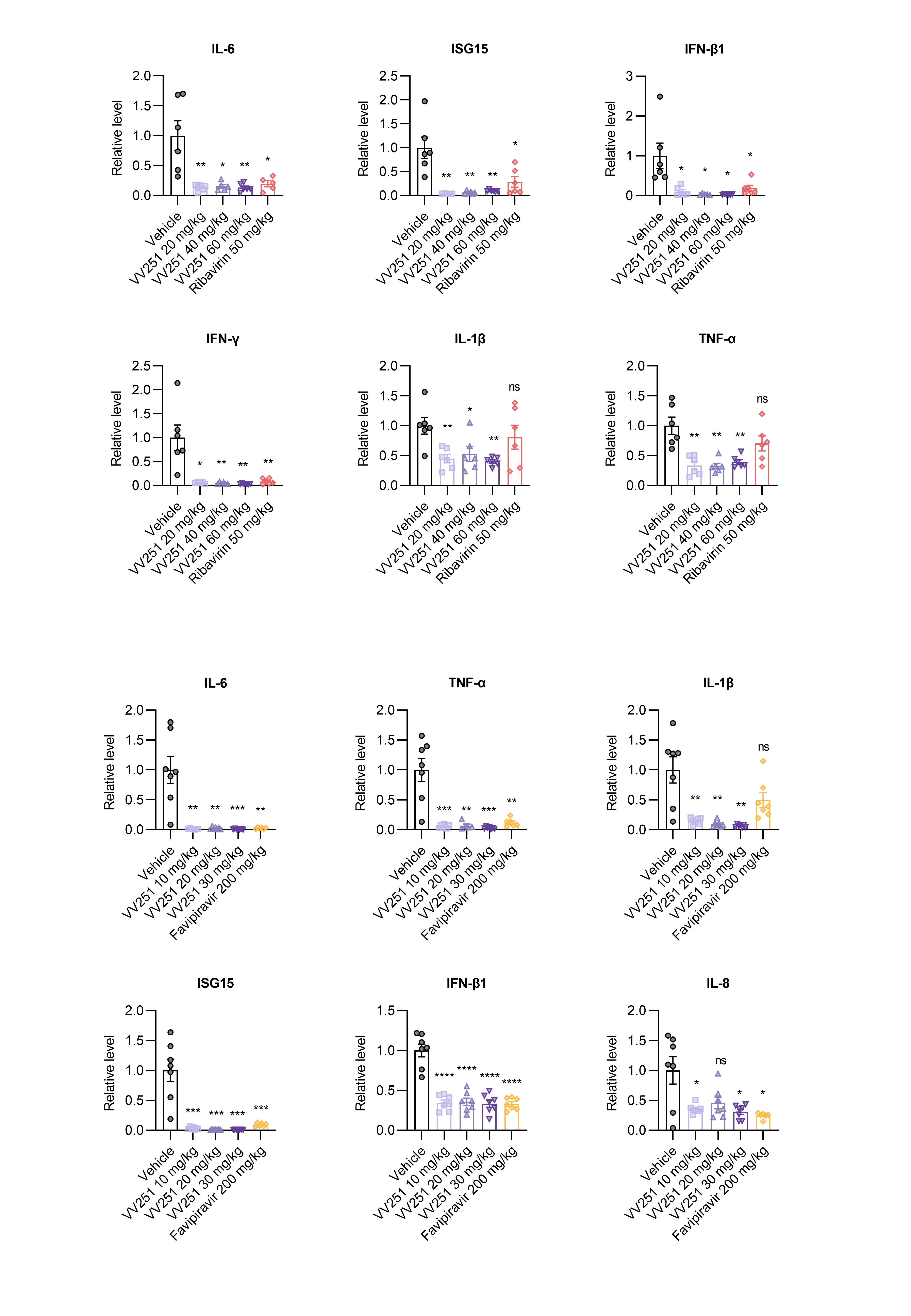


**FIG S6** Cytokine gene expression was measured in the lungs of mice infected with IAV PR8 on day 4 (n=7). GAPDH gene was used as the internal reference gene. The symbols represent individual values, and the error bars indicate the SEM. The ROUT method in GraphPad was used to remove the outliers from the data, and Q was set to 1%. Statistical significance compared to vehicle group was analysed by unpaired Student’s t test. **P* < 0.05; ***P* < 0.01; ****P* < 0.001; *****P* < 0.0001; and ns, not significant.


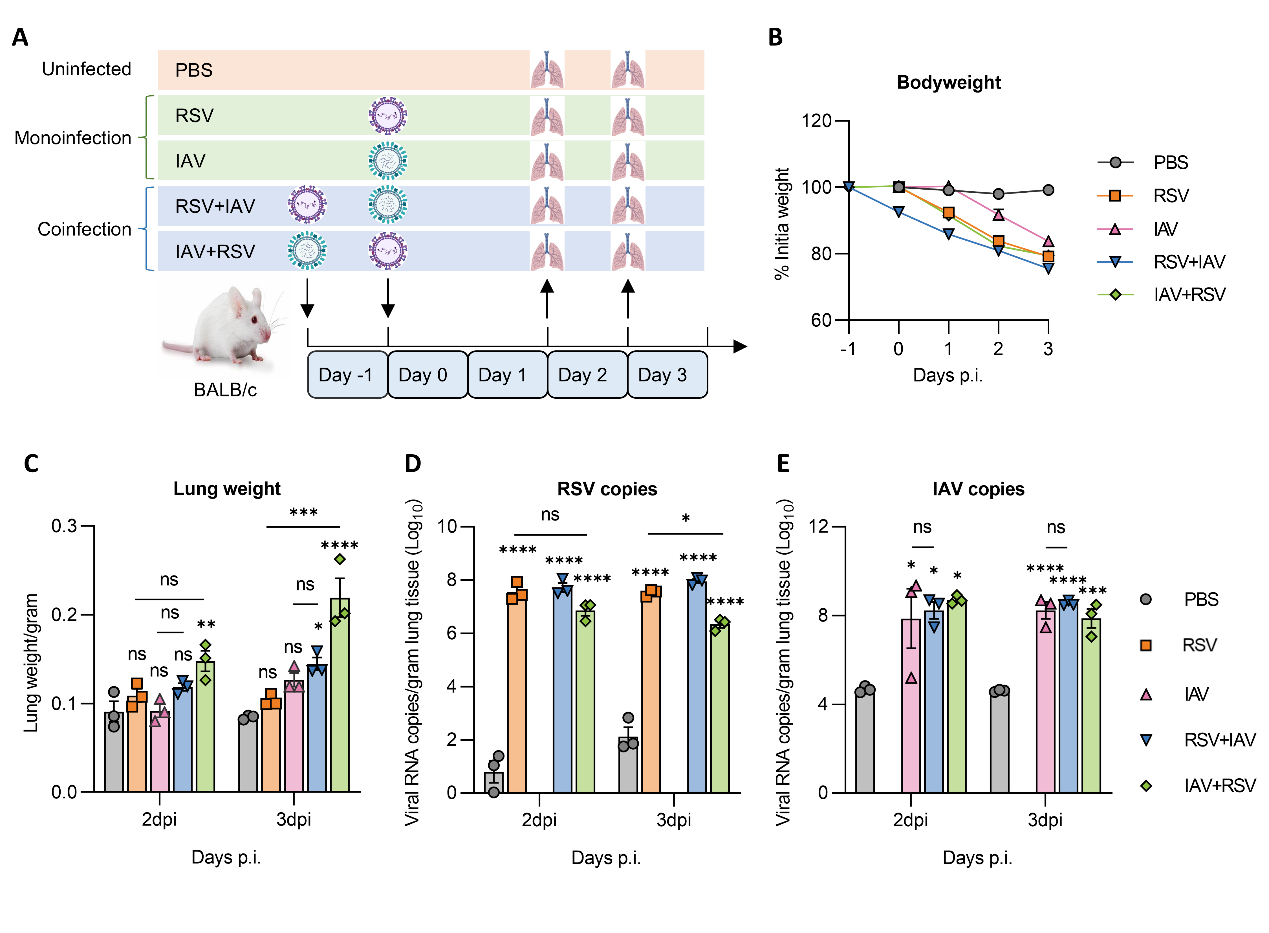


**FIG S7** Establishment of a mouse model for coinfection with RSV and IAV. (A) Schematic illustration of the coinfection protocol with RSV and IAV in BALB/c mice (n=3). (B) Body weight changes of mice following viral infection. (C) Lung tissue weight. (D) RSV viral levels in lung tissue. (E) IAV viral levels in lung tissue. The symbols represent individual values, and the error bars indicate the SEM. Statistical significance was analysed by one-way ANOVA tests. Symbol at the right top of column indicates the comparison to PBS group. **P* < 0.05; ***P* < 0.01; ****P* < 0.001; *****P* < 0.0001; and ns, not significant.


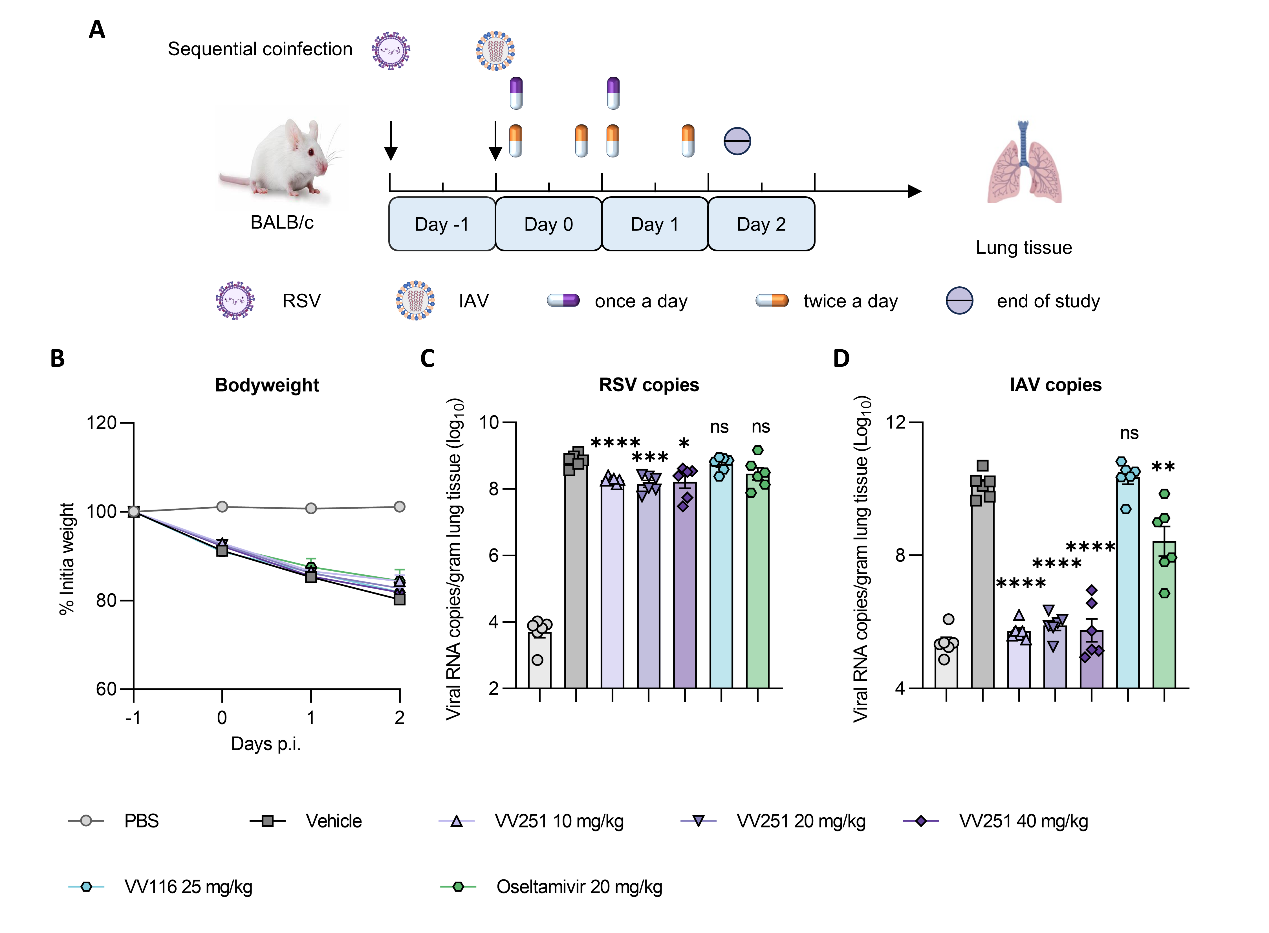


**FIG S8** Efficacy of VV251 in sequential RSV/IAV coinfection mouse model. (A) Schematic of the efficacy study in BALB/c mice (n=6). Experiments employed 6-8-week-old female BALB/c mice, which were intranasally coinfected with RSV and IAV sequentially, followed by once daily oral administration of VV251. (B) Shown is the percentage change in body weight compared with the starting body weight of BALB/c mice infected with RSV A2 and IAV PR8 sequentially (n=6). (C) and (D) Viral RNA levels in the lung tissue of PBS-, vehicle-, VV251- and ribavirin-treated mice on day 2 post-coinfection (n=6). The symbols represent individual values, and the error bars indicate the SEM. Statistical significance compared to vehicle group was analysed by unpaired Student’s t test. **P* < 0.05; ***P* < 0.01; ****P* < 0.001; *****P* < 0.0001; and ns, not significant.


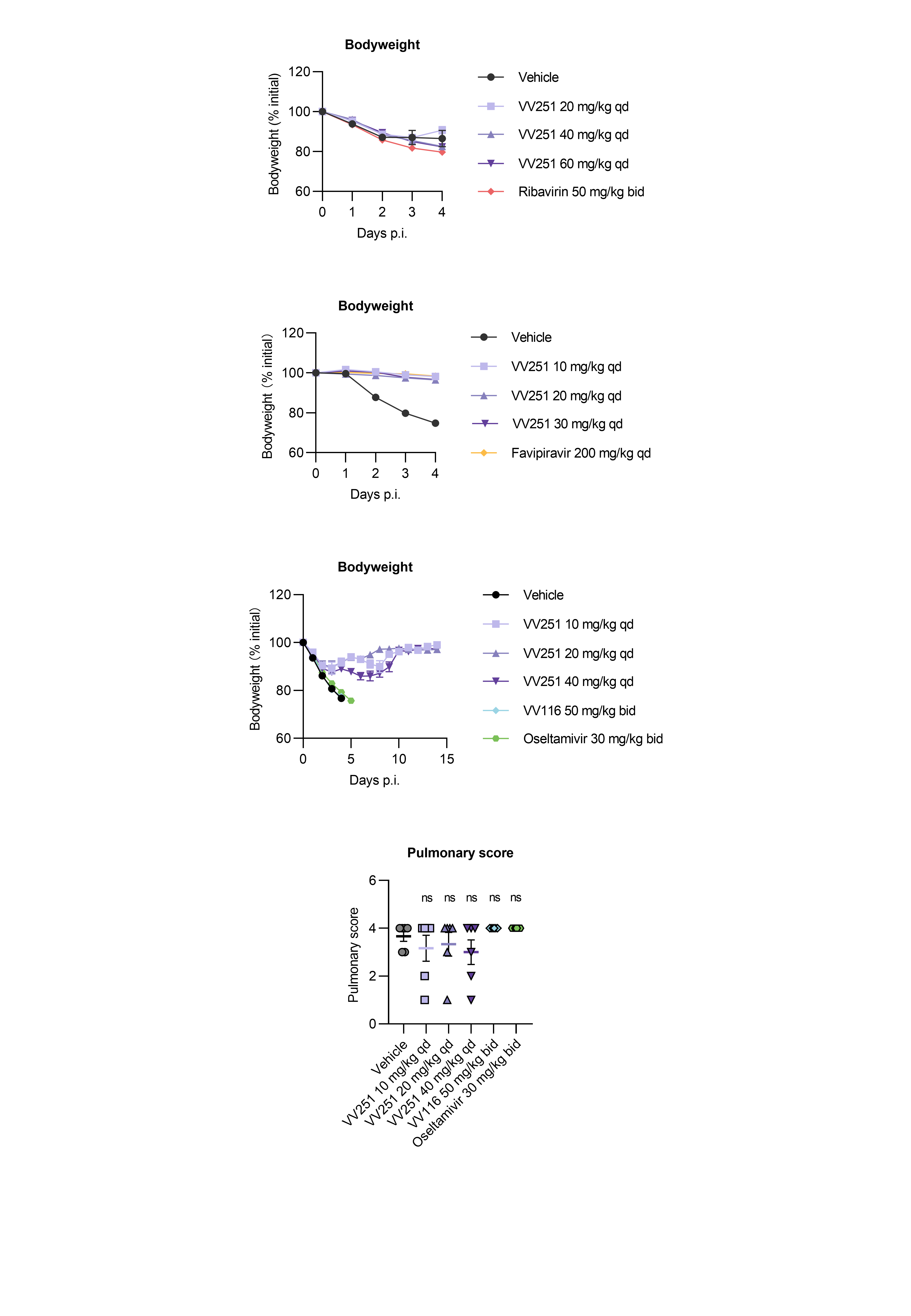


**FIG S9** Body weight changes in all five treatment groups and control animals after infection (n=6). The symbols represent the means ± SEM.


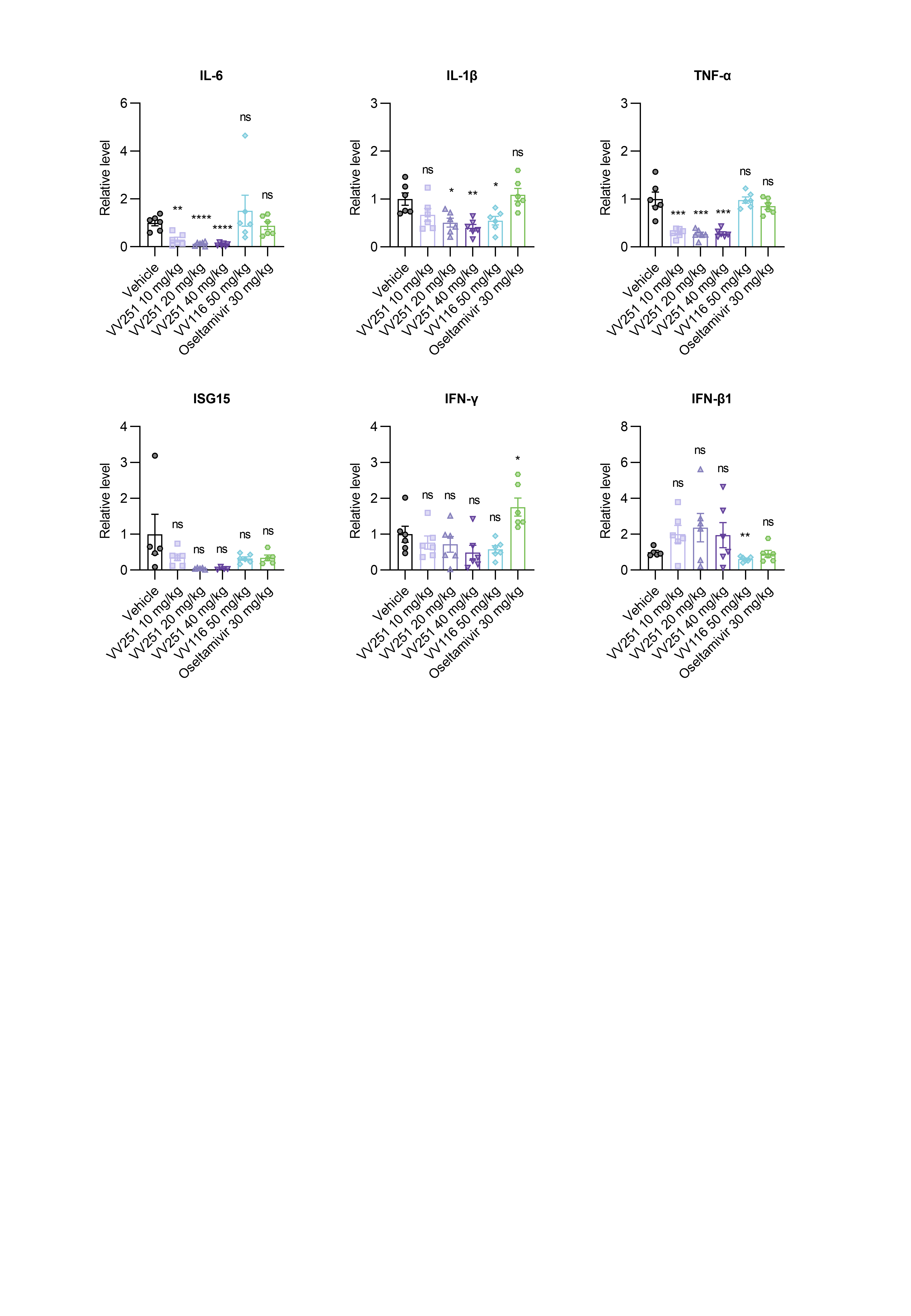


**FIG S10** Cytokine gene expression was measured in the lungs of mice infected with RSV and IAV simultaneously on day 4 (n=6). GAPDH gene was used as the internal reference gene. The symbols represent individual values, and the error bars indicate the SEM. The ROUT method in GraphPad was used to remove the outliers from the data, and Q was set to 1%. Statistical significance compared to vehicle group was analysed by unpaired Student’s t test. **P* < 0.05; ***P* < 0.01; ****P* < 0.001; *****P* < 0.0001; and ns, not significant.


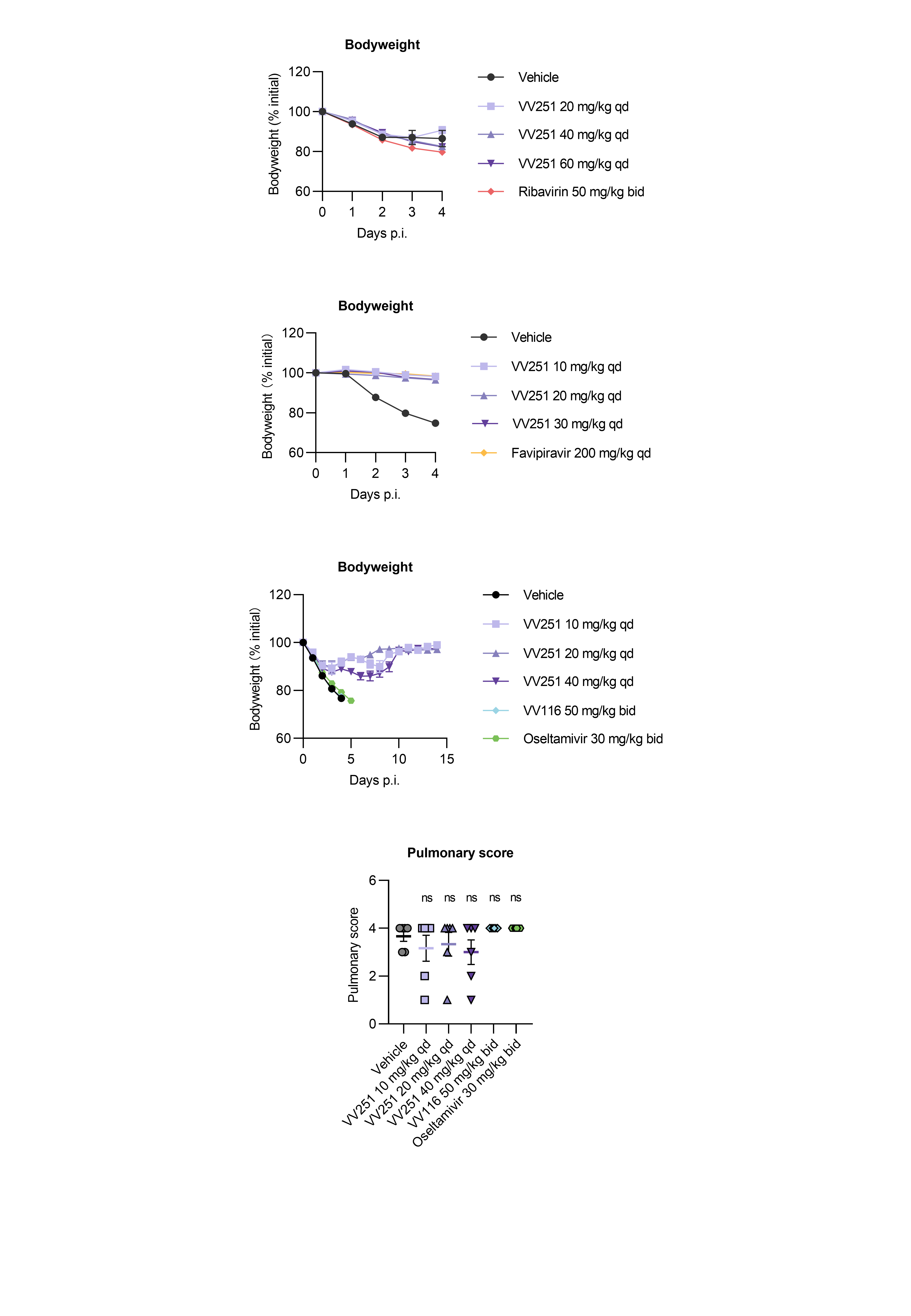


**FIG S11** Lung tissue pathological scores were evaluated at 4 dpi in drug-treated mice and control mice (n=6). Each symbol represents data for one mouse, and the line represents the mean. Statistical significance compared to vehicle group was analysed by unpaired Student’s *t* test. **P* < 0.05; ***P* < 0.01; ****P* < 0.001; *****P* < 0.0001; and ns, not significant.


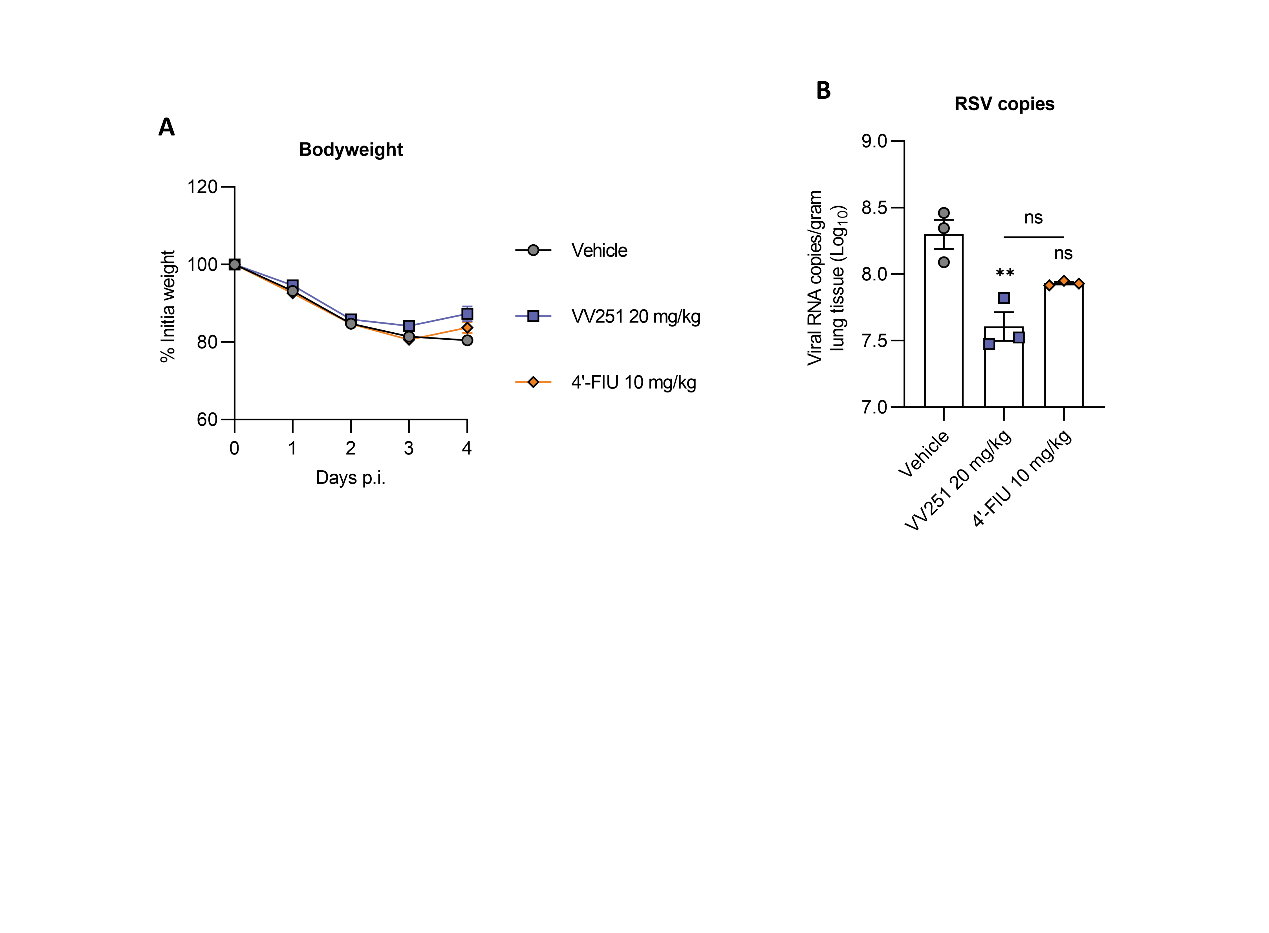


**FIG S12** Comparative antiviral activity of VV251 and 4′-FlU against RSV at equimolar doses. All data are presented as mean ± SEM. Statistical significance was analysed by one-way ANOVA tests. Symbol at the right top of column indicates the comparison to the vehicle group. **P* < 0.05; and ns, not significant.
